# Adaxial–abaxial leaf surface asymmetry is a key ecological driver of the phyllosphere microbiome

**DOI:** 10.1093/jxb/erag255

**Published:** 2026-05-29

**Authors:** Hiroki Sugimoto, Akinobu Toyoda, Yoshikazu Furuta, Tatsuya Iwase, Ritsuko Yaokawa, Ritsuko Kitagawa-Yogo, Masakazu Ito, Satoshi Katahira

**Affiliations:** Toyota Central R&D Labs., Inc., Nagakute, Aichi 480-1192, Japan; Frontier Research Center, Toyota Motor Corporation, Toyota, Aichi 471-8572, Japan; Toyota Central R&D Labs., Inc., Nagakute, Aichi 480-1192, Japan; Toyota Central R&D Labs., Inc., Nagakute, Aichi 480-1192, Japan; Toyota Central R&D Labs., Inc., Nagakute, Aichi 480-1192, Japan; Toyota Central R&D Labs., Inc., Nagakute, Aichi 480-1192, Japan; Frontier Research Center, Toyota Motor Corporation, Toyota, Aichi 471-8572, Japan; Toyota Central R&D Labs., Inc., Nagakute, Aichi 480-1192, Japan; Universität zu Köln, Germany

**Keywords:** Abaxial, adaxial, bacteria, ecosystem, epiphyte, fungi, microbiome, phyllosphere

## Abstract

Microorganism-driven ecosystems on leaf surfaces play pivotal roles in regulating plant health and fitness. While individual leaves provide distinct microhabitats on their adaxial (upper) and abaxial (lower) surfaces, most studies have assessed phyllosphere microbial community assembly and function using whole leaves, thereby overlooking their inherent spatial heterogeneity. Here, we independently analyzed bacterial and fungal communities on adaxial and abaxial leaf surfaces across seven temperate tree species over a 6-month period. We further assessed their metabolic and ecological profiles to understand how leaf surface compartmentalization shapes microbial communities. Our results revealed that compositionally distinct microbial communities were consistently established on each leaf surface. Co-occurrence network analyses showed that the abaxial communities exhibited differences in interaction structure compared with the adaxial communities, characterized by a higher proportion of negative correlations. Predicted functional profiles indicated that adaxial communities were enriched in degradation pathways, potentially reflecting nutrient-poor, high-stress conditions, whereas abaxial communities were enriched in biosynthetic and energy-generating functions, consistent with resource-rich environments. Collectively, our results highlight adaxial–abaxial leaf surface asymmetry as a key ecological driver shaping the structure, function, and interaction dynamics of the phyllosphere microbiome.

## Teaser

This study integrates bacterial and fungal community analyses to characterize leaf-side differences in interaction networks and predicted functional potential, highlighting combined effects of leaf surface traits and local microclimatic conditions.

## Introduction

Plants host diverse microbial communities across various ecological niches, collectively known as the phyllosphere (above-ground) and rhizosphere (below-ground) (Bulgarelli *et al*., 2013; Vacher *et al*., 2016). The phyllosphere, encompassing leaves, stems, flowers, buds, fruits, and seeds, harbors microorganisms both on its surfaces and within its internal structures. Leaves represent one of the largest and most complex microbial habitats on Earth (Vorholt, 2012). Leaf surfaces support diverse microorganisms, including bacteria, fungi, yeasts, viruses, and protozoa, which inhabit these environments either transiently or throughout their life cycles (Vacher *et al*., 2016; Shakir *et al*., 2021). Bacterial densities on leaf surfaces can reach 10^6^–10^7^ cells cm^−2^ (Lindow and Brandl, 2003), highlighting the leaf surface as a critical interface for interactions among microbes and between microbes and their host plants. These interactions play crucial roles in plant health and productivity (Ritpitakphong *et al*., 2016; Abadi *et al*., 2020; Legein *et al*., 2020; Liu *et al*., 2020) and contribute to broader ecosystem functioning (Laforest-Lapointe *et al*., 2017). Beyond plant-associated effects, leaf microbiomes are increasingly recognized for their roles in the phylloremediation of air pollutants and in shaping human immune system development (Mills *et al*., 2017; Wei *et al*., 2017; Maurya *et al*., 2023). Epiphytic communities may also serve as important sources of atmospheric microorganisms that disperse to built environments, with potential implications for human and indoor plant health (Bowers *et al*., 2011; Berg *et al*., 2014; Miletto and Lindow, 2015; Lymperopoulou *et al*., 2016; Toyoda *et al*., 2023). Consequently, leaf-associated microbial communities are of significant agricultural, ecological, and public health interest.

Leaf surfaces present challenging environments for microbial survival and colonization. The plant cuticle, a hydrophobic extracellular layer covering leaf surfaces, limits water and nutrient availability by acting as a barrier to the transport of water and polar substances (Schönherr, 2006; Beattie, 2011). Furthermore, water and nutrients are heterogeneously distributed across leaf surfaces (Schönherr, 2006; Beattie, 2011), with bacterial colonization often occurring at sites where these resources are more readily available, such as the base of trichomes, stomata, anticlinal epidermal cell walls, and grooves along veins (Leveau and Lindow, 2001; Remus-Emsermann *et al*., 2011). Leaf surfaces are also frequently exposed to fluctuations in temperature, humidity, and UV radiation. Consequently, epiphytic microorganisms have evolved diverse adaptive strategies, including pigment production for protection against UV irradiation (Jacobs *et al*., 2005) and the secretion of biosurfactants, such as syringafactin, to retain water and acquire nutrients from host tissues (Burch *et al*., 2014; Hernandez and Lindow, 2019).

Plants present heterogeneous habitats for microorganisms. Above-ground tissues—including leaves, buds, stems, branches, trunks, and flowers—function as distinct microhabitats that influence microbial community composition and diversity (Ottesen *et al*., 2013). Consequently, plant-associated microbial community assembly is shaped by the interaction of environmental heterogeneity and selective forces (Shakir *et al*., 2021). Accumulating evidence indicates that a variety of biotic and abiotic factors influence bacterial and fungal community assemblages on leaves, including host plant identity, geography, and seasonal variation (Lambais *et al*., 2006; Whipps *et al*., 2008; Redford and Fierer, 2009; Cordier *et al*., 2012; Kembel *et al*., 2014; Agler *et al*., 2016; Laforest-Lapointe *et al*., 2016; Espenshade *et al*., 2019). Structural and chemical features of leaf surfaces, such as trichome density and levels of water, soluble sugars, phosphorus, phenolics, and waxes, also affect bacterial colonization and population size (Tang *et al*., 2023). Therefore, the combination of leaf surface heterogeneity and external environmental fluctuations constitutes a selective pressure shaping epiphytic microbial community composition and diversity.

Extensive research on microbial community assembly and function on leaf surfaces has often treated the entire surface as a uniform habitat. However, leaves represent distinct environmental habitats on their adaxial and abaxial sides, despite their close proximity. This asymmetry arises from differences in cell morphology and physicochemical features (Jeffree, 2006; Schlechter *et al*., 2019). Trichome and stomatal densities frequently differ between the adaxial and abaxial surfaces (Werker, 2000; Watts *et al*., 2024), and surface hydrophobicity varies due to differences in cuticular wax microstructures (Li *et al*., 2017) and epicuticular wax composition across plant species (Gniwotta *et al*., 2005; Jeffree, 2006). Consistent with this, Arabidopsis mutants with altered cuticle or wax biosynthesis exhibit distinct bacterial community compositions, whereas trichome mutations alone have minimal effect (Reisberg *et al*., 2012, 2013; Ritpitakphong *et al*., 2016). Despite this evidence for strong habitat filtering, few studies have examined microbial assembly and function on adaxial and abaxial surfaces independently.

A recent study has shown that leaf-side asymmetry influences bacterial community assembly, with communities on adaxial leaf surfaces more strongly shaped by dispersal processes, whereas those on abaxial surfaces are predominantly structured by host plant filtering (Smets *et al*., 2023). However, leaves harbor diverse microbial groups, including fungi, on their surfaces, and it remains unclear whether such leaf-side differences extend to these microorganisms. Moreover, if contrasting assembly processes operate among microbial groups, then their ecological implications for leaf surface microbial communities remain to be clarified.

In this study, we complement and extend this framework by independently characterizing surface-specific bacterial and fungal communities on leaves from seven temperate tree species over a 6 month growing season. We examined intra- and inter-kingdom interaction structure, as well as predicted bacterial metabolic profiles and fungal ecological traits, aiming to determine how these features reflect habitat-specific community assembly on adaxial and abaxial leaf surfaces.

## Materials and methods

### Study site and plant sample collection

Tree leaf samples were collected from three subsites within the Forest of Toyota, located 6.25 km from downtown Toyota City in Aichi Prefecture, Japan: Tsubaki-Valley [35°2′11.4″N, 137°11′17.1″E; 57.8 m above sea level (asl)], Akebi-Trail (35°2′14.9″N, 137°11′19.5″E; 59.8 m asl), and Kojii-Hill (35°2′23.3″N, 137°11′17.5″E; 103.8 m asl) (Supplementary Fig. S1A). These subsites are situated at distances ranging from 125 m to 367 m from each other (Supplementary Fig. S1A). The forest is situated in a humid subtropical climatic zone. Climate data for 2020 were obtained from the Japan Meteorological Agency (JMA; https://www.jma.go.jp/jma/indexe.html) (Supplementary Fig. S1B, C). The Toyota meteorological station, JMA (35°7.9′N, 137°10.6′E, elevation 75 m asl), recorded an annual mean temperature of 16.0 °C and precipitation of 1696.0 mm in 2020. This station is located 10.48 km from the forest.

Mature leaves were collected monthly from July to December 2020 from nine trees representing seven native species (*Gamblea innovans*, *Quercus glauca*, *Prunus grayana*, *Castanopsis cuspidata*, *Ilex pedunculosa*, *Eurya japonica*, and *Ilex macropoda*) located at three subsites (Supplementary Table S1). The ages of the sampled trees were not determined because core sampling for age estimation was not performed. Two individuals each of *G. innovans* and *Q. glauca* were sampled, while the remaining species were represented by a single individual tree (Supplementary Table S1). At each sampling event, 3–9 fully developed leaves, located 1–2 m above the ground and appearing healthy with no visible necrosis or damage, were collected from each individual tree. During leaf sampling, γ-ray-sterilized rubber gloves were worn, and leaf sampling was performed carefully to avoid direct contact with the leaf surfaces. Each sampled leaf was individually wrapped in polyethylene film to secure its position and prevent cross-contamination between the adaxial and abaxial surfaces. The wrapped leaves were placed in sterile polyethylene bags, kept in a cool container during transport to the laboratory, and stored at 4 °C. Processing to dislodge epiphytic microorganisms from the leaves was conducted the day after sampling, as detailed in the following section.

### Leaf trait analysis

Leaf area was determined from whole-leaf scans using a photocopier and analyzed using ImageJ software (https://imagej.net/ij/). Stomatal density was measured using leaf surface molds/impressions, positioned at the center of each of three equal segments along the longitudinal axis of the leaf, as well as at the central positions of the main vein and leaf margin. Molds were prepared by applying the hydrophilic vinyl polysiloxane impression material EHAHIFLEX (GC Corporation, Tokyo, Japan) to three points on each side of the leaf and allowing it to harden. Each mold was then coated with colorless nail polish, removed using transparent cellophane tape, and mounted on a glass slide for light microscopic observation. Leaf surfaces were additionally observed using SU3500 and TM3000 scanning electron microscopes (Hitachi) at accelerating voltages of 10 kV and 15 kV, respectively.

### Microbial DNA extraction

Large dust particles were carefully removed from leaf surfaces using sterile, non-wet HydraFlock 6″ (15.24 cm) flock swabs with polystyrene handles (Puritan Medical Products, Guilford, ME, USA). To dislodge epiphytic microorganisms, the adaxial and abaxial surfaces of these pre-treated leaves were swabbed separately using sterile flock swabs pre-moistened with a swab solution (1× phosphate-buffered saline, pH 7.4, 1.0% Triton X-100, 0.5% Tween-20). Swabs from multiple leaves (3–9 leaves per sample) were combined to generate a single composite sample for each leaf surface. Swab tips were then cut directly into bead tubes supplied with the DNeasy PowerWater Kit (QIAGEN, Hilden, Germany) and stored at −80 °C until DNA extraction. Microbial DNA was subsequently extracted using the DNeasy PowerWater Kit, following the manufacturer’s instructions. For a negative control, DNA was extracted from unused sterile swab tips following the same extraction procedure as for the leaf samples to monitor potential contamination from swabs or reagents.

### Quantitative real-time PCR

Microbial DNA from each leaf surface was subjected to real-time PCR amplification using the GeneAce SYBR qPCR Mix α Low ROX (Nippon Gene, Tokyo, Japan) with the primers listed in Supplementary Table S2 to quantify bacterial 16S rRNA and fungal 28S rRNA genes. Thermal cycling parameters were set as follows: 95 °C for 10 min, followed by 45 cycles of 95 °C for 30 s and 60 °C for 1 min. Standard curves were generated using a dilution series of ZymoBIOMICS Microbial Community DNA standard (Zymo Research, Irvine, CA, USA), which contains 12% genomic DNA from eight bacterial species (*Pseudomonas aeruginosa*, *Escherichia coli*, *Salmonella enterica*, *Lactobacillus fermentum*, *Enterococcus faecalis*, *Staphylococcus aureus*, *Listeria monocytogenes*, and *Bacillus subtilis*) and 2% genomic DNA from two fungal species (*Saccharomyces cerevisiae* and *Cryptococcus neoformans*). Quantification was performed using a 7500 Real-Time PCR System (Applied Biosystems, Foster City, CA, USA) and 7500 Software for 7500 and 7500 Fast Real-Time PCR systems (Applied Biosystems). Bacterial and fungal DNA quantity per cm^2^ of leaf area was then calculated.

### Amplicon sequencing analysis

For bacterial and fungal identification, microbial DNAs extracted from each surface of the tree leaves were used to amplify the V1–V2 region of bacterial 16S rRNA genes and the fungal internal transcribed spacer 2 (ITS2) region using the primers listed in Supplementary Table S2. PCR was performed as follows: an initial hot start at 95 °C for 3 min, followed by 25 cycles at 95 °C for 30 s, 55 °C for 30 s, and 72 °C for 30 s, and a final extension at 72 °C for 5 min. The amplified PCR products were purified using the AMPure XP system (Beckman Coulter, Brea, CA, USA). A second round of PCR to attach Nextera XT indices and sequencing adapters (Illumina, San Diego, CA, USA) was performed as follows: an initial hot start at 95 °C for 3 min, followed by eight (bacterial 16S rRNA) or 10 (fungal ITS2) cycles at 95 °C for 30 s, 55 °C for 30 s, and 72 °C for 30 s, and a final extension at 72 °C for 5 min. The libraries were then normalized and pooled, and paired-end sequencing (bacteria, 2×150 sequencing cycles; fungi, 2×300 sequencing cycles) was performed on an Illumina iSeq system (Illumina) for bacterial 16S rRNA gene data and an Illumina MiSeq system (Illumina) for fungal ITS2 sequence data.

### Bioinformatics and statistical analyses

Each sample read was pre-processed, quality filtered, and analyzed using QIIME2 (bacteria, 2018.11; fungi, version 2022.8) (Bolyen *et al*., 2019). Initially, primer sequences were trimmed from bacterial and fungal demultiplexed FASTQ files using Cutadapt (v1.18) (Martin, 2011) and the FASTX-Toolkit (v0.0.14) (http://hannonlab.cshl.edu/fastx_toolkit/), respectively. The trimmed sequences were then denoised using the DADA2 software package (Callahan *et al*., 2016). Forward reads were used for bacterial 16S rRNA gene analysis because the read length generated by the iSeq system was insufficient to reliably merge paired-end reads spanning the V1–V2 region. For fungal ITS2 sequence analysis, merged paired reads were used. Taxonomic assignment was performed using the SILVA database (version 138) (Quast *et al*., 2013) for bacteria and the UNITE database (v9.0) (Kõljalg *et al*., 2013) for fungi.

Bacterial and fungal datasets were processed using the phyloseq (v1.42.0) (McMurdie and Holmes, 2013) and ggplot2 (v3.4.0) (Wickham, 2016) packages in R (version 4.2.2; R Core Team, 2016). Taxonomic classifications, amplicon sequence variants (ASVs), and metadata profiles were imported into the phyloseq object. To mitigate the effects of uneven sequencing depth and sampling bias (Schloss *et al*., 2011), bacterial and fungal sequences were rarefied to 22 966 and 71 955 reads per sample, respectively, based on the lowest read depth observed across all samples. Alpha diversity of microbial communities was assessed using ASV richness, Shannon diversity index, and Simpson diversity index. Statistical differences between groups were analyzed using the Wilcoxon rank-sum test.

Beta diversity was calculated using Bray–Curtis dissimilarity. Non-metric multidimensional scaling (NMDS) ordination was performed in three dimensions using the PRIMER package 7 software (v7.0.21). To determine the significance of the differences in microbial community composition between adaxial and abaxial leaf surfaces, permutational multivariate analysis of variance (PERMANOVA) (Anderson, 2017) was conducted using PRIMER 7 with the PERMANOVA+ add-on. As most tree species analyzed were represented by a single tree, host species and sampling month were not tested as independent explanatory factors. Leaf surface was treated as a fixed factor, while the combination of individual tree and sampling month was included only as a random factor to account for the paired adaxial–abaxial sampling structure. *P*-values were generated using 9999 permutations of residuals under a reduced model with type III (partial) sums of squares. The resulting *F* statistic is reported as pseudo-*F*, and the *P* statistic as *P*(perm). To evaluate homogeneity of variances between leaf surfaces, permutational analysis of multivariate dispersions (PERMDISP) was also conducted using 9999 permutations (Anderson, 2006). Furthermore, analysis of similarity (ANOSIM) (Clarke, 1993) based on Bray–Curtis dissimilarity was performed in the PRIMER 7 software using 9999 permutations to explore dissimilarities in microbial community composition between leaf surfaces.

Differentially abundant microbial taxa between adaxial and abaxial leaf surfaces were identified using Linear Discriminant Analysis Effect Size (LEfSe, v1.0.8) (Segata *et al*., 2011). Enriched taxa were defined as those with linear discriminant analysis scores >3.0. Similarity percentage (SIMPER) analysis was performed using PRIMER 7 to identify the taxa that contributed most to the differences between leaf surfaces. Core taxa on each surface were defined as taxa present in all adaxial or abaxial samples with >0.1% relative abundance, consistent with previous studies (Grady *et al*., 2019; Gao *et al*., 2021).

For genus-level co-occurrence network reconstruction, bacterial and fungal sequences were rarefied to 22 966 reads per sample. The Sparse Correlations for Compositional Data (SparCC) algorithm (Friedman and Alm, 2012) was employed with default parameter settings. *P*-values were calculated from 1000 bootstrapped datasets and adjusted using the Benjamini–Hochberg correction. SparCC correlations with an absolute magnitude >0.5 and statistically significant adjusted *P*-values (*P* < 0.05) were selected for microbial network construction and visualized using Gephi (v0.10.1) (Bastian *et al*., 2009).

Network robustness was evaluated using igraph (v1.3.4) (Csárdi and Nepusz, 2006) and ggplot2 (v3.4.0) (Wickham, 2016) packages in R (version 4.2.2; R Core Team, 2016). Two node-removal strategies, random node removal and targeted node removal based on node degree, were applied to SparCC-derived microbial interaction networks (Albert *et al*., 2000; Solé and Montoya, 2001; Dunne *et al*., 2002). For each removal step, the relative size of the largest connected component (LCC) was calculated as the size of the largest connected subnetwork divided by the number of nodes in the original network. For random node removal analysis, nodes were removed at increasing fractions, and each removal fraction was simulated 200 times. The area under the LCC-removal curve (AUC) was calculated for each simulation replicate, and differences between groups were analyzed using the Wilcoxon rank-sum test.

Bacterial functional prediction from 16S rRNA gene sequencing data was performed using Phylogenetic Investigation of Communities by Reconstruction of Unobserved States (PICRUSt2) (v2.3.0b) (Douglas *et al*., 2020). Bacterial functional profiles were subsequently inferred based on the abundance of metabolic pathways using the MetaCyc database (Caspi *et al*., 2020). For PERMANOVA of PICRUSt2-predicted metabolic pathway profiles, species-level averaged profiles were used. Leaf surface was treated as a fixed factor, while the combination of tree species and sampling month was included only as a random factor to account for the paired adaxial–abaxial sampling structure. Statistical comparisons of metabolic pathways between two groups were performed using Welch’s *t*-test with a 95% confidence interval in the statistical analysis of metagenomic profiles (STAMP) software (v2.1.3) (Parks *et al*., 2014). Benjamini–Hochberg-adjusted *P*-values < 0.05 were considered statistically significant. Fungal ecological traits were assigned at the genus level using the FungalTraits database (v1.2) (Põlme *et al*., 2020); when primary and secondary lifestyles differed, the primary lifestyle was selected.

Venn diagrams were generated using the Venn (v1.11) (Dusa, 2024) and VennDiagram (v1.7.3) (Chen and Boutros, 2011) packages in R. Boxplots were generated using standard settings: the box edges represent the 25th and 75th percentiles, the median is indicated by a line inside the box, the whiskers represent the most extreme non-outlier data points, and outliers are indicated by open circles.

## Results

### Leaf architecture varies among tree species and between the adaxial and abaxial surfaces of the same species

To investigate phyllosphere microorganisms on both leaf surfaces, samples were collected from seven temperate tree species—three deciduous (*G. innovans*, *P. grayana*, and *I. macropoda*) and four evergreen (*Q. glauca*, *C. cuspidata*, *I. pedunculosa*, and *E. japonica*)—over a 6 month period starting in July 2020 in Toyota, Aichi Prefecture, Japan (Supplementary Fig. S1; Supplementary Table S1). All sampled trees were hypostomatous, with stomata present exclusively on the abaxial surface (Supplementary Table S3), consistent with previous observations in most tree species (Grubb *et al*., 1975; Boeger *et al*., 2004). Given that stomatal density decreases as leaves expand, except during initial growth phases (Gay and Hurd, 1975; Schurr *et al*., 2000; Cardoso *et al*., 2018), we confirmed that all leaves had reached full size and maturity at the start of sampling (Supplementary Fig. S2).

SEM analysis of adaxial and abaxial leaf surfaces revealed distinct differences in surface configuration, trichome type, and trichome density (Supplementary Fig. S3; Supplementary Table S3). For example, *E. japonica* exhibited relatively smooth surfaces with no observable trichomes on either surface. Conversely, *Q. glauca* displayed sparse capitate glandular trichomes on the adaxial surface, while abaxial surfaces were densely covered with two types of trichomes: uniseriate solitary trichomes and bifurcate trichomes.

### Distinct structural and diversity patterns of bacterial and fungal communities on adaxial and abaxial leaf surfaces

Across most tree species, adaxial leaf surfaces generally exhibited higher microbial loads for both bacteria and fungi than abaxial surfaces (Supplementary Fig. S4). High-throughput amplicon sequencing yielded 6 418 125 bacterial 16S rRNA gene reads, and 14 288 127 fungal ITS reads, which were assigned to 1210 bacterial and 2493 fungal taxa at the lowest taxonomic rank, respectively.

**Fig. 1.**
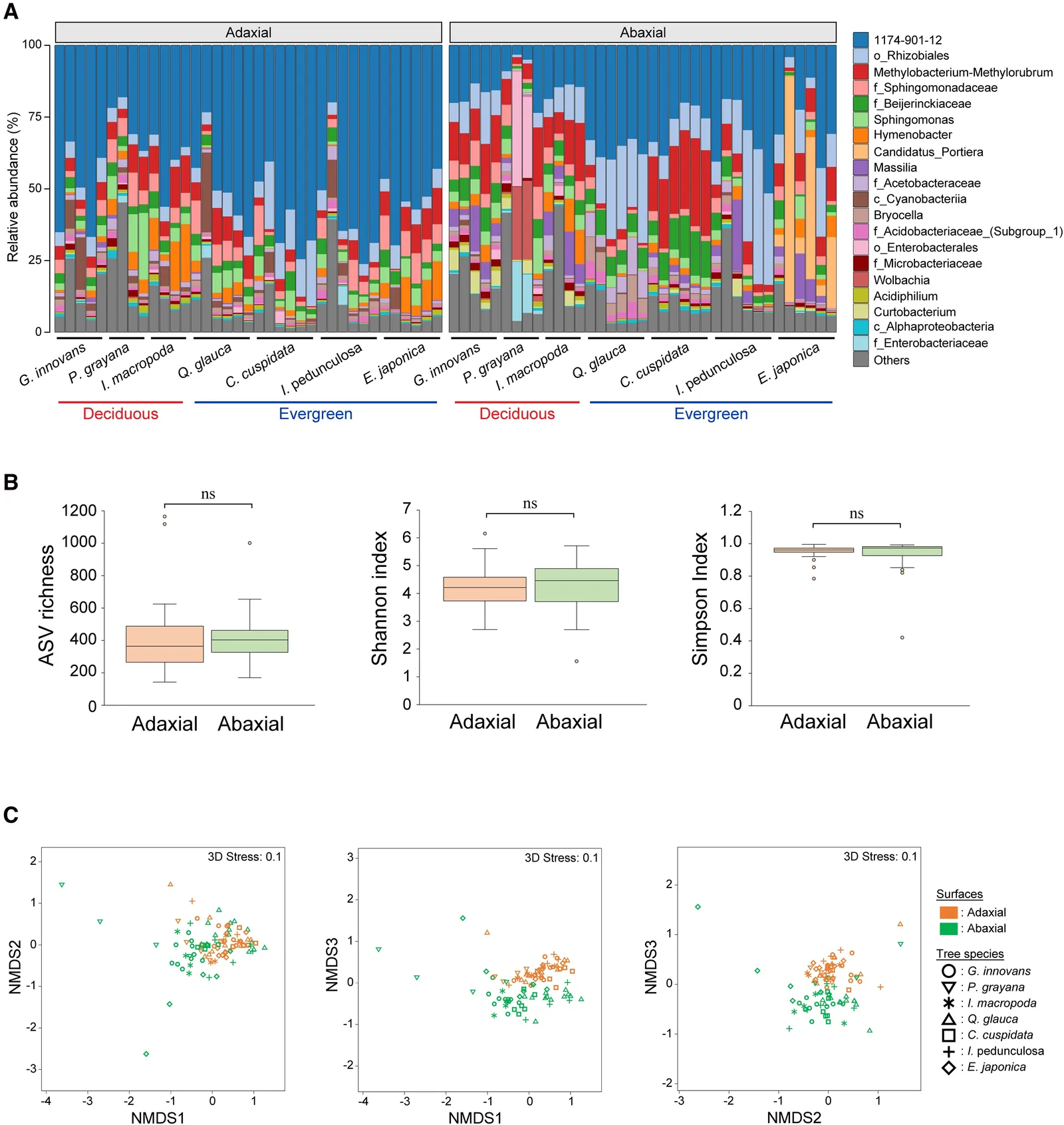
Bacterial community compositions and diversity on the adaxial and abaxial surfaces of tree leaves. (A) Relative abundance of bacterial taxa at the genus level (top 20). The order in each sample is July, August, September, October, November, and December. Samples for *G. innovans* could not be obtained in December, and those for *P. grayana* and *I. macropoda* were unavailable in November and December since these trees are deciduous species. (B) Bacterial alpha diversity. Each value is counted and calculated after rarefaction (ns, not significant; Wilcoxon rank-sum test). (C) Non-metric multidimensional scaling (NMDS) ordination in three dimensions using Bray–Curtis dissimilarity community composition at genus level. (Left) First and second axes. (Center) First and third axes. (Right) Second and third axes. Each point corresponds to a sample, with the different colors representing leaf surfaces, and the different shapes representing tree species.

**Fig. 2.**
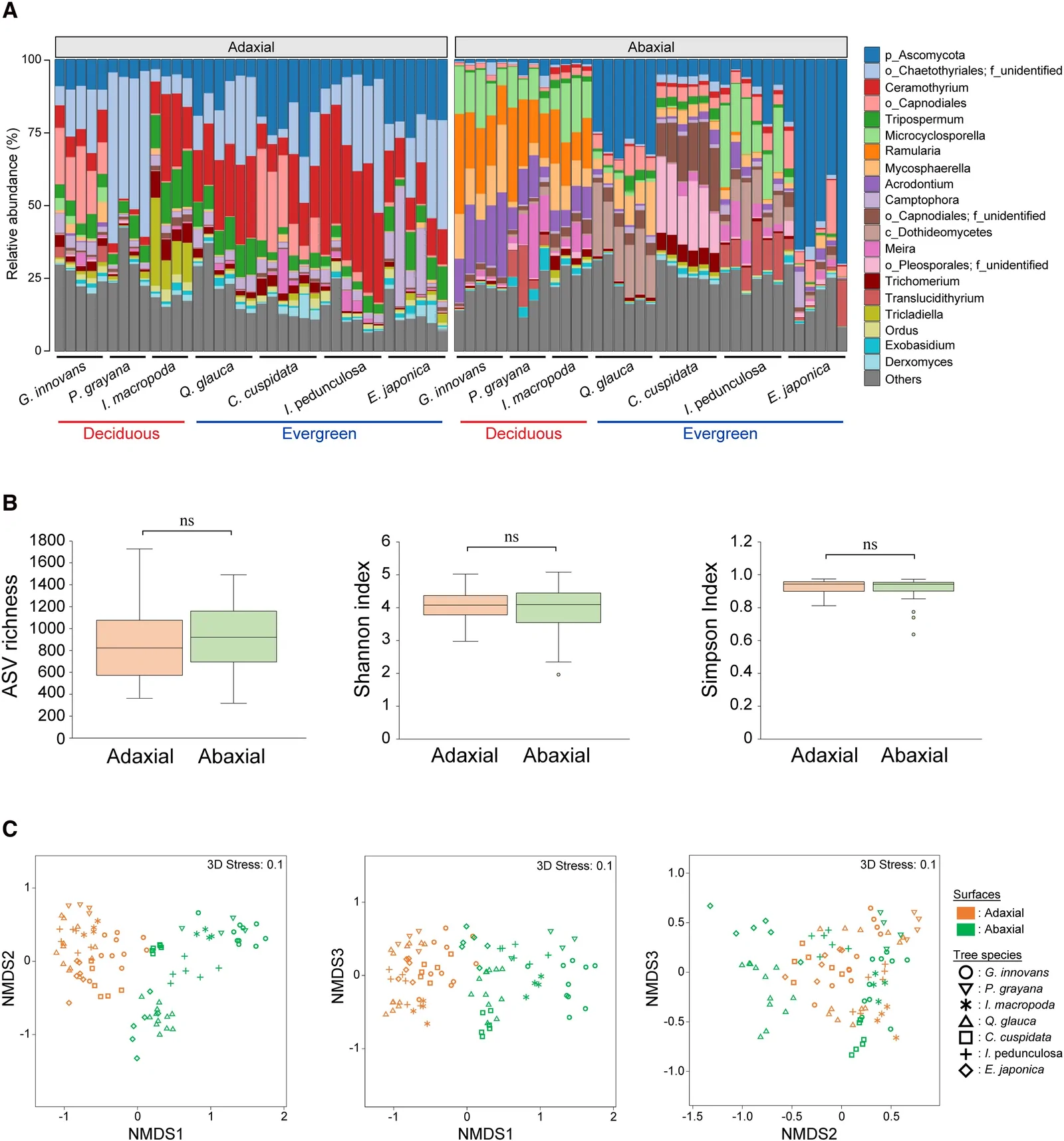
Fungal community compositions and diversity on the adaxial and abaxial surfaces of tree leaves. (A) Relative abundance of fungal taxa at the genus level (top 20). The order in each sample is July, August, September, October, November, and December. Samples for *G. innovans* could not be obtained in December, and those for *P. grayana* and *I. macropoda* were unavailable in November and December since these trees are deciduous species. (B) Fungal alpha diversity. Each value is counted and calculated after rarefaction (ns, not significant; Wilcoxon rank-sum test). (C) Non-metric multidimensional scaling (NMDS) ordination in three dimensions using Bray–Curtis dissimilarity community composition at genus level. (Left) First and second axes. (Center) First and third axes. (Right) Second and third axes. Each point corresponds to a sample, with the different colors representing leaf surfaces, and the different shapes representing tree species.

Taxonomic profiling revealed distinct differences in microbial community structure between adaxial and abaxial leaf surfaces (Figs 1A, 2A; Supplementary Figs S5, S6). Bacterial communities on adaxial (96.8%) and abaxial (98.0%) leaf samples were dominated by five phyla: *Proteobacteria*, *Bacteroidota*, *Actinobacteriota*, *Acidobacteriota*, and *Cyanobacteria* (Supplementary Fig. S5A). Within *Proteobacteria*, *Gammaproteobacteria* were more abundant on abaxial surfaces, whereas *Alphaproteobacteria* were common to both surfaces (Supplementary Fig. S5B). At the genus level, *1174-901-12* was the most dominant taxon across both surfaces (Fig. 1A). Representative sequences of *1174-901-12* were manually inspected using BLAST against the National Center for Biotechnology Information nucleotide database, confirming their bacterial origin.

Fungal communities were primarily composed of *Ascomycota* on both adaxial (90.6%) and abaxial (89.2%) leaf surfaces, followed by *Basidiomycota* (8.3% and 10.7%, respectively) (Supplementary Fig. S6A). At the class level, community composition differed, with *Eurotiomycetes* predominating on adaxial surfaces and *Dothideomycetes* on abaxial surfaces (Supplementary Fig. S6B). The most abundant taxa on adaxial surfaces included *Chaetothyriales* (unidentified family), *Ceramothyrium*, and *Tripospermum*. Abaxial surfaces exhibited host-dependent variation: in deciduous trees, genera such as *Ramularia*, *Acrodontium*, *Microcyclosporella*, and *Mycosphaerella* were prevalent, whereas evergreen species were rich in *Capnodiales*, *Pleosporales*, and other *Dothideomycetes*.

No significant differences in alpha diversity (ASV richness, Shannon index, and Simpson index) were observed between surfaces for either bacterial or fungal communities (Figs 1B, 2B). A substantial proportion of microbial taxa were shared between the two surfaces at both the lowest identifiable taxonomic level and the genus level (bacteria, 47% at the lowest identifiable taxonomic level and 53% at the genus level, and fungi 53% and 64%, respectively; Supplementary Fig. S7).

PERMANOVA of beta diversity indicated significant differences in both bacterial and fungal community composition between adaxial and abaxial leaf surfaces, with leaf surface accounting for 13.7% and 27.2% of the variation in bacterial and fungal communities, respectively (Tables 1, 2). Although dispersion differed significantly between adaxial and abaxial samples (bacteria, *F* = 17.290, *P* = 0.0003; fungi, *F* = 46.882, *P* = 0.0001), NMDS ordinations demonstrated distinct clustering by leaf side. This separation occurred along the third NMDS axis for bacteria and the first axis for fungi (Figs 1C, 2C). ANOSIM confirmed significant surface-related differences, with fungal communities exhibiting stronger dissimilarity between surfaces (*R* = 0.701, *P* < 0.01) than bacterial communities (*R* = 0.304, *P* < 0.01).

**Table 1.** PERMANOVA assessing the effect of leaf surface on genus-level bacterial community composition

Source	df	SS	MS	Pseudo-*F*	*R* ^2^	*P*(perm)
Leaf surface	1	16 402	16 402	18.506	0.137	0.0001
Residuals	47	41 657	886.32			
Total	95	119 300				

Genus-level compositional dissimilarity was based on the Bray–Curtis dissimilarity index. The combination of individual tree and sampling month was included as a random factor to account for the paired adaxial–abaxial sampling structure but was not interpreted as an explanatory factor. df, degrees of freedom; SS, sum of squares; MS, mean squares; Pseudo-*F*, pseudo-*F* ratio; *P*(perm), permutation *P*-value. *P*(perm) was obtained using 9999 permutations.

**Table 2.** PERMANOVA assessing the effect of leaf surface on genus-level fungal community composition

Source	df	SS	MS	Pseudo-*F*	*R* ^2^	*P*(perm)
Leaf surface	1	63 521	63 521	40.834	0.272	0.0001
Residuals	47	73 114	1555.6			
Total	95	233 130				

Genus-level compositional dissimilarity was based on the Bray–Curtis dissimilarity index. The combination of individual tree and sampling month was included as a random factor to account for the paired adaxial–abaxial sampling structure but was not interpreted as an explanatory factor. df, degrees of freedom; SS, sum of squares; MS, mean squares; Pseudo-*F*, pseudo-*F* ratio; *P*(perm), permutation *P*-value. *P*(perm) was obtained using 9999 permutations.

Differential abundance analyses (LEfSe and SIMPER) identified taxa contributing to surface-specific structuring of microbial communities. Specifically, the bacterial genus *1174-901-12* and the fungal genera *Ceramothyrium* and *Tripospermum* significantly contributed to compositional differentiation between adaxial and abaxial leaf surfaces (Supplementary Figs S8, S9; Supplementary Tables S4, S5).

Overall, leaf surface was a significant factor shaping the structure and composition of both bacterial and fungal communities.

### Adaxial–abaxial leaf surface environments develop distinct microbial co-occurrence networks

The distinct microbial communities inhabiting each leaf surface facilitated the identification of core microbial taxa, defined as genera with >0.1% relative abundance consistently present across all host species and sampling times. Nine and eight core bacterial taxa were identified on adaxial and abaxial surfaces, respectively (Supplementary Fig. S10A; Supplementary Table S6). Seven taxa were shared between the two surfaces, including *1174-901-12*, *Methylobacterium-Methylorubrum*, and *Sphingomonas*, with *1174-901-12* being the most abundant. Similarly, 11 and eight core fungal taxa were detected on adaxial and abaxial surfaces, respectively, with four shared taxa: *Tripospermum*, *Chaetothyriales*, *Capnodiales*, and *Ascomycota* (Supplementary Fig. S10B; Supplementary Table S7). These results indicate surface-specific developmental processes within microbial communities despite the overlap in core members, suggesting distinct functional and ecological roles.

**Fig. 3.**
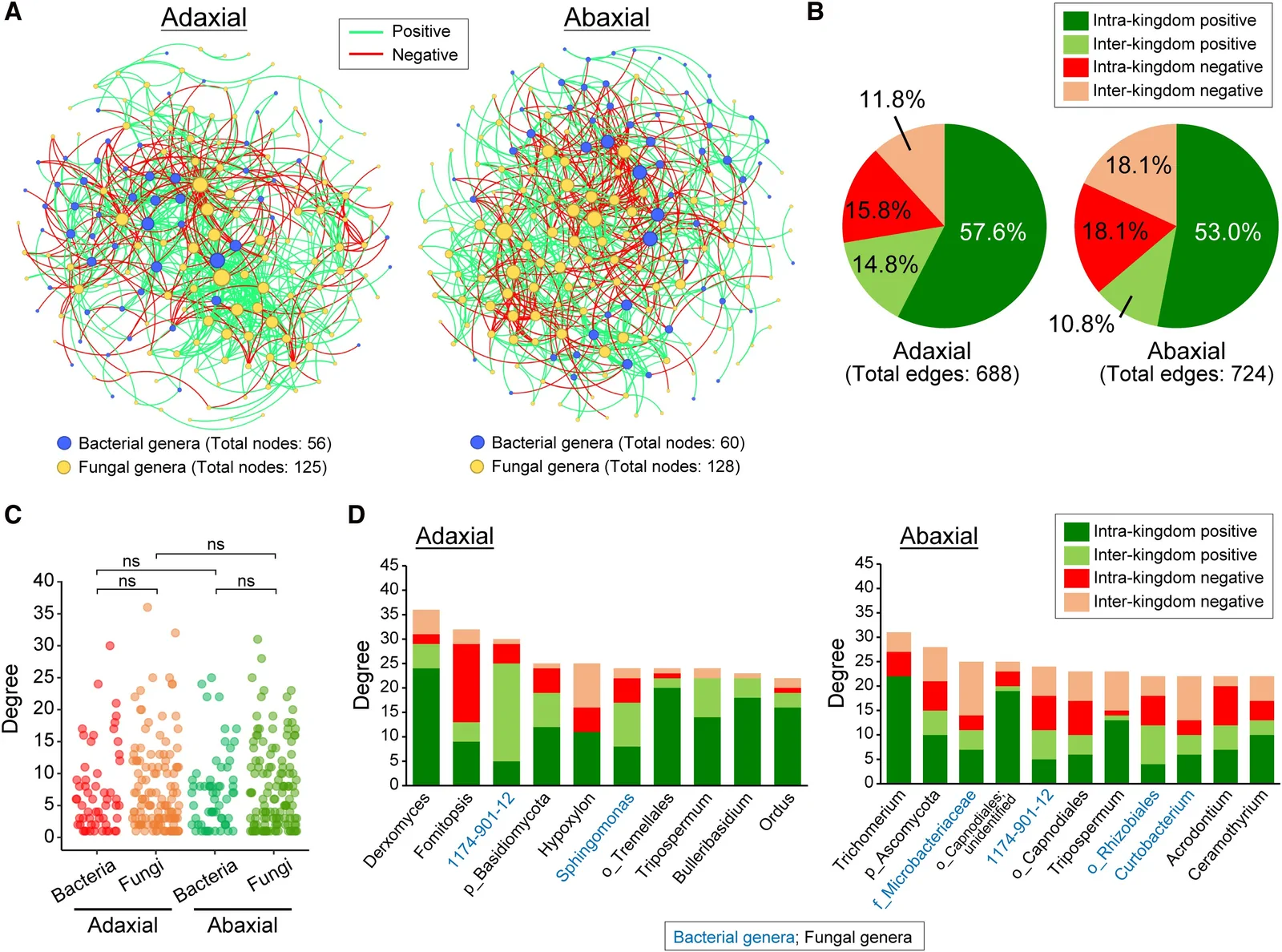
Inter-kingdom co-occurrence networks on leaf surfaces. (A) Inter-kingdom co-occurrence networks on adaxial and abaxial surfaces of all trees. The node color represents bacterial (blue) and fungal (yellow) genera. The node size depends on the degree of connection. The edge color shows positive (light green) and negative (red) correlations. (B) Proportion of intra-kingdom (bacterial–bacterial and fungal–fungal) and inter-kingdom positive and negative correlations in microbial networks on adaxial and abaxial surfaces. (C) Degree values of bacterial and fungal genera in microbial co-occurrence networks on leaf surfaces (ns, not significant; Wilcoxon rank-sum test). (D) Degree and correlation type of the top 10 nodes in the microbial networks.

To investigate microbial hierarchical interactions on adaxial and abaxial leaf surfaces, we constructed intra-kingdom (bacterial–bacterial and fungal–fungal) and inter-kingdom (bacterial–fungal) co-occurrence networks (Fig. 3; Supplementary Figs S11, S12). Intra-kingdom networks on both surfaces displayed comparable complexity with respect to node and edge counts, as well as node degree distributions (Supplementary Figs S11A, C, S12A, C). Although both microbial networks exhibited a skewed node degree distribution, characterized by a limited number of highly connected nodes and a larger proportion of nodes with few connections, fungal networks contained more nodes and edges than bacterial networks (Supplementary Figs S11A, C, S12A, C). The majority of intra-kingdom edges were positive (Supplementary Figs S11B, S12B). Notably, bacterial networks on the abaxial surface showed a greater proportion of negative correlations compared with the adaxial surface (Supplementary Fig. S11B), with a similar trend observed in the fungal networks, albeit to a lesser extent (Supplementary Fig. S12B). The top 10 hub nodes in abaxial bacterial networks demonstrated more negative correlations than their adaxial counterparts (Supplementary Fig. S11D). Conversely, fungal networks exhibited an opposing pattern, displaying a greater number of negative edges among the top 10 fungal hubs on the adaxial surface (Supplementary Fig. S12D). Core bacterial genera constituted approximately half of the top 10 hub nodes in bacterial networks, whereas core fungal genera were under-represented among the top fungal hubs (Supplementary Figs S11D, S12D; Supplementary Tables S6, S7).

In inter-kingdom networks, node and edge numbers, as well as degree distributions, were comparable between adaxial and abaxial leaf surfaces (Fig. 3A, C). Fungal nodes outnumbered bacterial nodes in both adaxial and abaxial networks, including among the top 10 hubs (Fig. 3A, C, D). Positive intra-kingdom correlations were predominant; however, negative correlations were more frequent in the abaxial inter-kingdom network (Fig. 3B). On abaxial surfaces, bacterial–fungal correlations accounted for half of all negative correlations, whereas on adaxial surfaces a slightly higher proportion of intra-kingdom negative correlations (57.2%) was observed. A similar pattern was observed among the top 10 hub nodes of both inter-kingdom networks (Fig. 3D). Several core bacterial taxa were identified among top hub nodes (adaxial, *Sphingomonas*; abaxial, *1174-901-12* and *o_Rhizobiales*; Fig. 3D; Supplementary Table S6). Conversely, numerous core fungal taxa were identified as the top inter-kingdom hubs (adaxial, *Derxomyces*, *Ordus*, and *p_Basidiomycota*; abaxial, *Acrodontium*, *Tripospermum*, *o_Capnodiales*, *o_Capnodiales*; *unidentified*, and *p_Ascomycota*; Fig. 3D; Supplementary Table S7).

We further assessed the robustness of inter-kingdom networks on adaxial and abaxial leaf surfaces using two node-removal strategies, random node removal and targeted node removal based on node degree (Supplementary Fig. S13). Both node-removal simulations showed broadly comparable robustness of the adaxial and abaxial networks, with the abaxial networks showing a slight tendency toward higher robustness. These findings indicate that while the overall network complexity was similar, abaxial surfaces exhibited a higher proportion of negative correlations, particularly in inter-kingdom interactions.

### Bacterial communities on adaxial and abaxial leaf surfaces exhibit distinct predicted metabolic functions

Given that microbial diversity and network complexity vary across leaf surfaces, we assessed the predicted functional potential of 16S rRNA bacterial communities using PICRUSt2 (Douglas *et al*., 2020). Overall, 434 metabolic pathways were inferred from bacterial communities on both surfaces. NMDS ordination using Bray–Curtis distances revealed a slight but distinct separation along the third NMDS axis between adaxial and abaxial leaf surfaces (Supplementary Fig. S14). PERMANOVA also confirmed a significant surface-related difference in predicted metabolic pathway profiles, accounting for 12.0% of the variation (pseudo-*F* = 15.706, *P* = 0.0001; PERMDISP, *F* = 1.672, *P* = 0.2517).

**Fig. 4.**
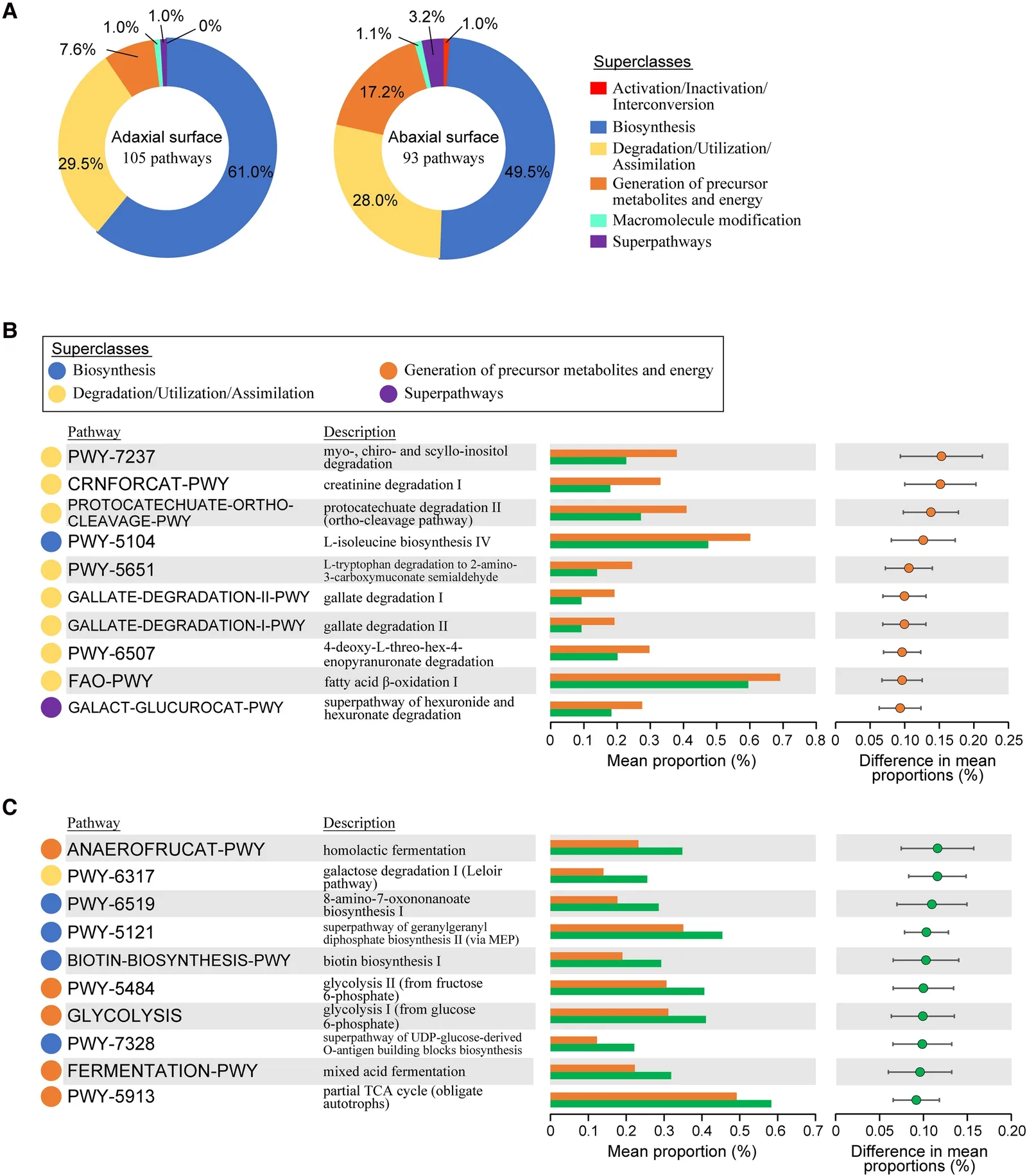
Differential functional profiles of bacterial communities between the adaxial and abaxial sides of leaf surfaces. Bacterial metabolic pathways on the adaxial and abaxial leaf surfaces were predicted using PICRUSt2. (A) Pie chart showing a superclass classification of metabolic pathways significantly enriched in bacterial communities on the adaxial (left) and abaxial (right) surfaces. See also Supplementary Table S10. (B, C) Relative abundance differences of the top 10 bacterial metabolic pathways enriched on the adaxial (B) and abaxial (C) surfaces. Bar plots on the left side represent significantly differentially abundant metabolic pathways of bacterial communities between the adaxial and abaxial leaf surfaces. Orange and green bars indicate pathways enriched on adaxial and abaxial leaf surfaces, respectively. Each pathway is labeled as a superclass category. Dot plots on the right side represent differences in mean proportions of metabolic pathways detected on both leaf sides with 95% confidence intervals. Pairwise comparisons between these two pathways are performed using Welch’s *t*-test with Benjamini–Hochberg correction (*P* < 0.05). Further details are provided in Supplementary Tables S8 and S9.

Among the predicted pathways, 198 pathways exhibited differential abundance between adaxial and abaxial surfaces. Of these, 105 and 93 pathways were significantly enriched in adaxial and abaxial bacterial communities, respectively (Supplementary Tables S8, S9). Most pathways were associated with three top-level functional categories: biosynthesis, degradation/utilization/assimilation, and generation of precursor metabolites. Notably, abaxial bacterial communities harbored a higher proportion of pathways for the generation of precursor metabolites (17.2%) than those on the adaxial surface (7.6%) (Fig. 4A; Supplementary Table S10).

On the adaxial surface, enriched pathways were predominantly classified into the degradation/utilization/assimilation category. These were assigned to second-level categories such as amine and polyamine degradation (CRNFORCAT-PWY); amino acid degradation (PWY-5651); aromatic compound degradation (GALLATE-DEGRADATION-I-PWY; GALLATE-DEGRADATION-II-PWY; PROTOCATECHUATE-ORTHO-CLEAVAGE-PWY); and secondary metabolite degradation (PWY-6507, PWY-7237) (Fig. 4B; Supplementary Table S8). In contrast, abaxial pathways were enriched in biosynthesis and generation of precursor metabolites, including carbohydrate biosynthesis (PWY-7328); cofactor, carrier, and vitamin biosynthesis (BIOTIN-BIOSYNTHESIS-PWY); fermentation (ANAEROFRUCAT-PWY, FERMENTATION-PWY); glycolysis (GLYCOLYSIS, PWY-5484); other biosynthesis (PWY-6519); secondary metabolite biosynthesis (PWY-5121); and tricarboxylic acid (TCA) cycle (PWY-5913) (Fig. 4C; Supplementary Table S9). Taken together, these results indicate that bacterial communities on adaxial and abaxial leaf surfaces exhibit distinct predicted metabolic pathway profiles.

### Ecological functions of fungal communities are determined by adaxial–abaxial differences

Similar to bacteria, fungal communities exhibit considerable taxonomic diversity and perform unique ecosystem functions (Põlme *et al*., 2020). The distinct taxonomic composition observed between adaxial and abaxial leaf surfaces prompted investigation into fungal community-level functions. Fungal taxa were classified based on ecological trait categories available in the FungalTraits database (Põlme *et al*., 2020).

**Fig. 5.**
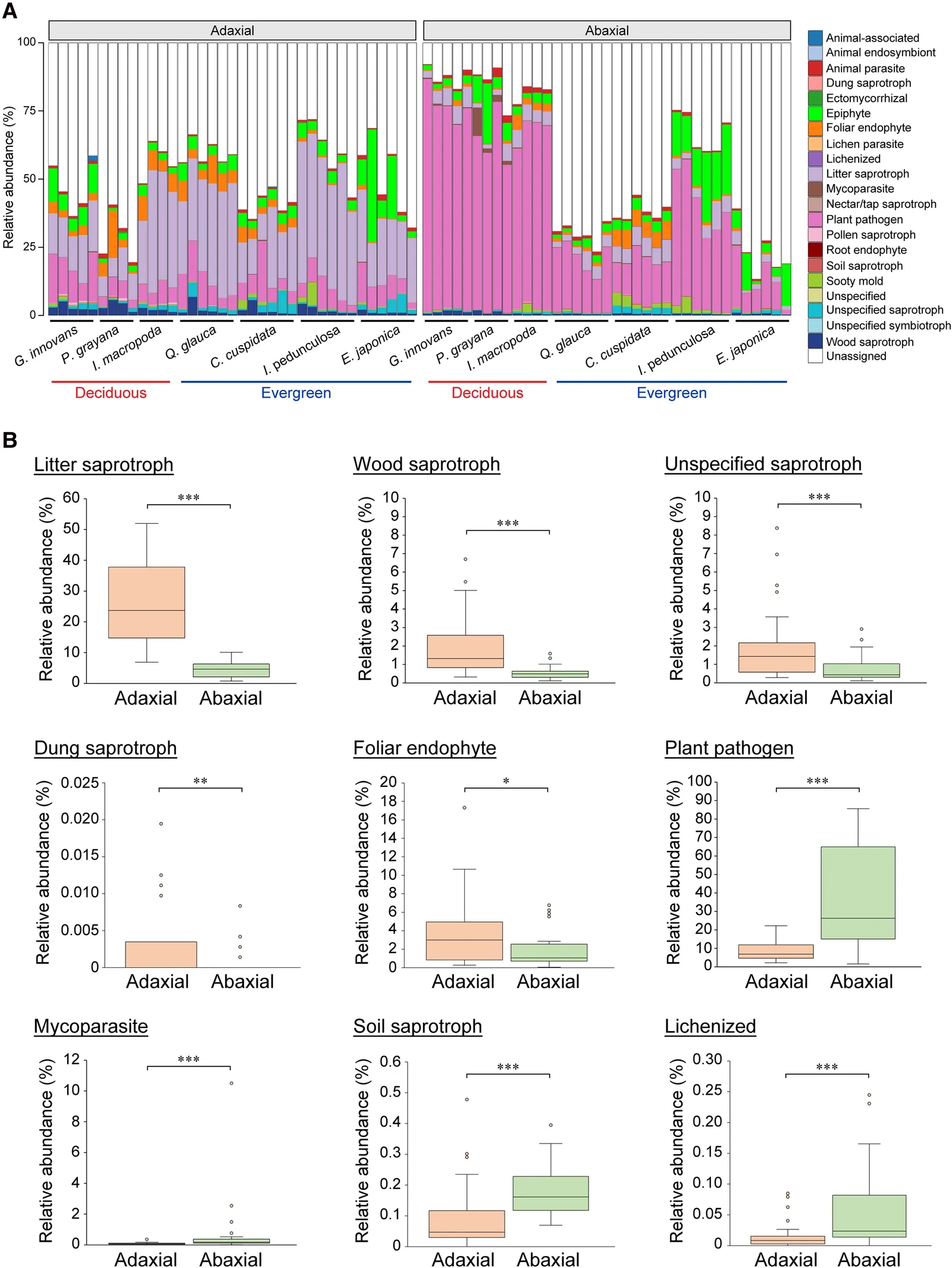
Differential ecological traits of fungal communities between adaxial and abaxial sides of leaf surfaces. (A) Ecological trait compositions of fungal communities on leaves (top 20). The order in each sample is July, August, September, October, November, and December. Samples for *G. innovans* could not be obtained in December, and those for *P. grayana* and *I. macropoda* were unavailable in November and December since these trees are deciduous species. (B) Differential abundance of fungal ecological traits between adaxial and abaxial leaf surfaces (***P < 0.001; **P < 0.01; *P < 0.05; Wilcoxon rank-sum test adjusted using Benjamini–Hochberg correction).

Of the 1280 fungal taxa examined, ecological traits could be assigned to 950 genera. Adaxial leaf surfaces were primarily dominated by saprotrophic fungi, including those categorized as Litter Saprotrophs, Wood Saprotrophs, Unspecified Saprotrophs, or Dung Saprotrophs, as well as foliar endophytic fungi (Fig. 5). Litter Saprotrophs represented the most abundant trait category (Fig. 5B). In contrast, the abaxial leaf surface exhibited significantly higher proportions of fungi classified as Plant Pathogens, Mycoparasites, Soil Saprotrophs, and Lichenized fungi (Fig. 5B). Notably, Plant Pathogen was the most prevalent ecological trait on the abaxial surface, irrespective of host type (Fig. 5A), despite marked differences in fungal composition between deciduous and evergreen hosts (Fig. 2).

These findings suggest that adaxial–abaxial leaf surface compartments are key determinants influencing fungal ecological behaviors, thereby shaping their functional roles within host-associated ecosystems.

## Discussion

In this study, bacterial and fungal community structure and composition differed significantly between adaxial and abaxial leaf surfaces. These findings highlight adaxial–abaxial leaf surface compartmentalization as a key factor shaping microbial communities and surface-specific metabolic and ecological differentiation.

Functional predictions based on PICRUSt2 should be interpreted with caution, as they infer metabolic potential from phylogenetic information rather than directly measuring gene content or expression. The contrasting patterns observed between adaxial and abaxial leaf surfaces may reflect differences in local environmental constraints specific to each leaf surface, such as nutrient availability, exposure to atmospheric deposition, or microclimatic stressors. However, these environmental factors were not directly measured in this study and should be interpreted as potential drivers rather than confirmed mechanisms. These patterns are consistent with the framework proposed by Smets *et al.* (2023), who suggest that bacterial communities on abaxial leaf surfaces are predominantly shaped by host filtering, whereas those on adaxial surfaces are more strongly influenced by dispersal and immigration processes, based on their analyses across 24 different plant species. In this context, the predicted functional differences in bacterial communities may represent leaf side-specific community assembly under these contrasting selective pressures. Thus, leaf-side effects may be associated with the combined influences of leaf surface traits and local microclimatic conditions.

On the adaxial leaf surface, bacterial communities exhibited an enrichment of metabolic pathways associated with the degradation of diverse compounds, including amino acids, aromatic compounds, lipids, nucleotides, and secondary metabolites (Supplementary Table S10). The observed enrichment in nucleotide metabolism may be associated with bacterial stress tolerance mechanisms, such as the modulation of cell wall properties (Solopova *et al*., 2016) and biofilm formation (Garavaglia *et al*., 2012). Conversely, on the abaxial leaf surface, bacterial communities were enriched in biosynthetic and energy-generating pathways, including aminoacyl-tRNA charging, amine and polyamine biosynthesis, polyprenyl biosynthesis, secondary metabolite biosynthesis, and tetrapyrrole biosynthesis (Supplementary Table S10), suggesting the conditions that may be associated with more favorable, nutrient-rich microhabitats.

Analysis of the top 10 significantly enriched pathways highlighted further functional differences between bacterial communities. On adaxial surfaces, the most enriched pathway was myo-, chiro-, and scyllo-inositol degradation. Given that inositol has been reported as an available carbon source on leaf surfaces (Ryffel *et al*., 2016; Flores-Núñez *et al*., 2020), enrichment of inositol degradation pathways on adaxial surfaces may indicate differential utilization of available substrates. Furthermore, bacterial communities on adaxial surfaces were enriched in pathways related to plant cell wall degradation, producing pyruvate and 3-keto-adipate from pectin- and lignin-derived compounds, such as gallate and protocatechuate, which are subsequently processed via glycolysis and the TCA cycle for energy production (Kasai *et al*., 2004; Kamimura *et al*., 2017; Cai *et al*., 2021).

Conversely, abaxial bacterial communities exhibited an enrichment of biosynthetic and energy-generating pathways, including biotin biosynthesis, glycolysis, the TCA cycle, and fermentation. Biotin, crucial for fatty acid biosynthesis, TCA cycle function, and amino acid metabolism (Streit and Entcheva, 2003), is an auxotrophic requirement for many leaf-associated bacteria (Bai *et al*., 2015; Ryback *et al*., 2022). These auxotrophic bacteria readily participate in metabolic cross-feeding interactions, the exchange of essential metabolites between two or more different strains, which contribute to the maintenance of community diversity and stability (Ryback *et al*., 2022). Notably, the observed enrichment of fermentation pathways potentially indicates an energy-efficient strategy known as overflow metabolism. In this process, bacteria preferentially utilize fermentation, a more protein-efficient process, over the higher energy-yielding respiration process, optimally allocating limited proteome resources even under aerobic conditions when high growth rates are favored (Basan *et al*., 2015). These predicted functional differences may indicate that bacterial communities on the abaxial leaf surface exhibit relatively higher biosynthetic and energy-efficient metabolic potential. Conversely, those on the adaxial surface may be associated with a greater representation of pathways for degradation of complex organic matter, probably originating from atmospheric deposition, under relatively constrained environmental conditions. Direct metagenomic or transcriptomic analyses are required to validate these functional inferences.

In contrast to the functional annotation of bacterial communities, we characterized fungal communities on both leaf surfaces using ecological traits from the FungalTraits database (Põlme *et al*., 2020). Despite limitations in genus-level assignment for a subset of fungal ASVs, the ecological profiling provided valuable functional insights. On the adaxial surface, fungal communities were dominated by saprotrophic taxa, particularly those associated with litter and wood decomposition (Fig. 5). Members of the class *Eurotiomycetes* comprised nearly half of the fungal community on this surface (Supplementary Fig. S6B), with a large majority of these taxa belonging to the order *Chaetothyriales*, most of which are saprobes (Tian *et al*., 2021) (Supplementary Fig. S6C). Notably, three saprobic fungal genera—*Ceramothyrium* (Yen *et al*., 2018), *Tripospermum* (Patil *et al*., 2019), and *Derxomyces* (Liu *et al*., 2012)—were among the top 20 most abundant taxa and significantly contributed to community differentiation (Fig. 2A). Conversely, the fungal communities on the abaxial surfaces were dominated by members of the class *Dothideomycetes*, particularly the order *Capnodiales* (Supplementary Fig. S6B, C) (Ohm *et al*., 2012; Abdollahzadeh *et al*., 2020). This class includes four *Capnodiales* genera: *Acrodontium* (Videira *et al*., 2016), *Microcyclosporella* (Frank *et al*., 2010), *Mycosphaerella* (Bakhshi and Braun, 2022), and *Ramularia* (Bakhshi *et al*., 2025). In addition, *Meira*, a genus within *Exobasidiomycetes*, is associated with plant pathogenesis (Yasuda *et al*., 2006). These fungal genera were among the top 20 most abundant taxa and were significantly more abundant on the abaxial surface (Fig. 2A). Some of these pathogenic fungi can enter leaf tissues through stomata on the leaf surface (Blodgett and Swart, 2002; Goodwin *et al*., 2011), whereas others can directly enter leaf tissues through the cuticle by differentiating specialized infection structures, such as appressoria (Asai and Shirasu, 2015). Overall, the functional profiles of fungal communities closely mirrored those of bacterial communities, indicating that both kingdoms undergo parallel adaptation to the distinct environmental and structural conditions of adaxial and abaxial leaf surfaces, consistent with the distinct assembly processes for bacterial communities on each leaf surface described by Smets *et al.* (2023).

Microorganisms on leaf surfaces form complex ecological interaction networks involving competition, facilitation, and inhibition (Vorholt, 2012; Agler *et al*., 2016; Chaudhry *et al*., 2021). Complex networks with higher connectivity are generally more resilient to environmental stresses than simpler ones (Santolini and Barabási, 2018), and cooperative and competitive interactions can influence ecological stability (Faust and Raes, 2012; Gao *et al*., 2021). Negative correlations, often indicative of competitive exclusion, have been suggested to stabilize communities and enhance ecosystem productivity more effectively than cooperative correlations (Oliveira *et al*., 2014; Coyte *et al*., 2015).

In this study, intra-kingdom network analysis revealed that fungal communities exhibited greater taxonomic diversity (nodes) and network complexity compared with bacterial communities on both leaf surfaces. Several factors may contribute to this pattern. First, many fungal species are closely associated with host plants, leading to more structured and diverse fungal networks (Aragón *et al*., 2017). Second, differences in marker gene resolution between fungi and bacteria due to sequencing strategies may affect taxonomic resolution and inferred network complexity.

Consistent with previous reports for leaf and root microbial networks in *Arabidopsis thaliana*, *Capsicum annuum*, *Sorghum bicolor*, and soil microbial networks (Agler *et al*., 2016; Durán *et al*., 2018; Gao *et al*., 2021, 2022), intra-kingdom correlations were mostly positive on both leaf sides. In contrast, 63% of inter-kingdom correlations on abaxial surfaces were negative, and this pattern was also reflected in the top 10 hub taxa (Fig. 3D). However, overall robustness patterns were comparable between leaf surfaces, indicating that differences in association patterns do not necessarily correspond to differences in network robustness.

According to the stress gradient hypothesis, microbial associations tend to be more positive under environmental stresses, such as drought or toxicity, which reduce competition and promote mutualistic interactions (Hoek *et al*., 2016; Piccardi *et al*., 2019; Gao *et al*., 2022). The adaxial surface represents a harsh environment, potentially favoring more positive microbial interactions, whereas on the abaxial surface, competitive interactions may arise from shared utilization of plant-derived carbohydrates, such as glucose, fructose, and sucrose (Leveau and Lindow, 2001; Ryffel *et al*., 2016). Spatial structuring and resource partitioning are known to enhance network stability (Oliveira *et al*., 2014; Coyte *et al*., 2015). Therefore, leaf surface traits, including age and volatile emissions, shape microbial composition and interactions. Similar to soil and rhizosphere systems, microbial diversity contributes to ecosystem functional redundancy and multifunctionality (Lindahl *et al*., 2007; Durán *et al*., 2018; Wagg *et al*., 2019), highlighting the importance of structural and functional diversity in maintaining phyllosphere ecosystem services.

In this study, consistently abundant microbial taxa were defined as core members, potentially playing central roles in microbial ecosystems shaped by leaf surface traits and local microenvironmental conditions. Notably, the bacterial genus *1174-901-12* (*Beijerinckiaceae*) and the fungal genus *Tripospermum* were core members on both surfaces, exhibiting stronger negative inter-kingdom correlations on abaxial surfaces (Fig. 3D). *1174-901-12* has been identified as a hub and a shared phyllosphere member across multiple tree species (Espenshade *et al*., 2019; Smets *et al*., 2023; Duan *et al*., 2024). *Tripospermum* is widely associated with plant hosts (Pem *et al*., 2019), and some members represent the asexual morph (anamorph) of *Trichomerium* (Hongsanan *et al*., 2016), influencing bacterial communities on leaf surfaces (Lv *et al*., 2023) and producing antifungal metabolites against *Alternaria* sp. (Haituk *et al*., 2022). Both taxa have been observed in non-plant environments, such as ceramic roof tiles and painted surfaces of buildings, suggesting roles as early colonizers in establishing microbial hubs (Shirakawa *et al*., 2002; Romani *et al*., 2019). As core microbial members are critical for optimal network and ecosystem functioning in host-associated microbiomes (Toju *et al*., 2018), further research is needed to elucidate their ecological contributions to microbial community structure and host interactions.

This study presents certain limitations. Plant age was not accounted for, the study duration was limited to 6 months, and the analyses focused solely on bacterial and fungal communities, thereby excluding viruses and other microorganisms. Future studies are necessary to address these limitations and to provide a more comprehensive understanding of the system.

In conclusion, microbial communities on adaxial and abaxial leaf surfaces exhibit distinct functional and ecological differentiation reflective of their respective environments. These findings underscore the necessity of treating each surface as a unique habitat within the phyllosphere. Future research on reciprocal interactions between microbial communities and host plants is essential to harness ecosystem services, including sustainable plant health (biocontrol), productivity (bioinoculant/biofertilizer), phylloremediation, and contributions to human immune system development.

## Supplementary Material

erag255_Supplementary_Data

## Data Availability

The datasets underlying this article are available in the DNA Data Bank of Japan (DDBJ, https://www.ddbj.nig.ac.jp) and can be accessed with DRR747133–DRR747228 and DRR747229–DRR747324.

## Supplementary data

The following supplementary data are available at *JXB* online.

Fig. S1. Sampling sites and climate conditions.

Fig. S2. Tree leaf (leaflet) parameters.

Fig. S3. Leaf epidermis of seven tree species under SEM.

Fig. S4. Microbial genomic DNA content on the adaxial and abaxial surfaces of tree leaves.

Fig. S5. Bacterial community compositions.

Fig. S6. Fungal community compositions.

Fig. S7. Venn diagrams of unique and shared microbial taxa between adaxial and abaxial leaf compartments, shown at the lowest taxonomic and genus levels.

Fig. S8. Differential abundance analysis of bacterial taxa between adaxial and abaxial sides of leaf surfaces.

Fig. S9. Differential abundance analysis of fungal taxa between adaxial and abaxial sides of leaf surfaces.

Fig. S10. Venn diagrams of microbial genera by host species.

Fig. S11. Bacterial intra-kingdom co-occurrence networks on leaf surfaces.

Fig. S12. Fungal intra-kingdom co-occurrence networks on leaf surfaces.

Fig. S13. Robustness analysis of inter-kingdom microbial interaction networks on adaxial and abaxial leaf surfaces.

Fig. S14. Non-metric multidimensional scaling ordination in three dimensions using Bray–Curtis dissimilarity of the predicted metabolic pathways of the bacterial communities on adaxial and abaxial sides of leaf surfaces.

Table S1. List of tree species and their order, family, and leaf habit.

Table S2. Primers used for quantitative real-time PCR and amplicon sequencing.

Table S3. Leaf epidermal features.

Table S4. Similarity percentage analysis assessing the contribution of specific bacterial genera to the dissimilarity between bacterial communities on adaxial and abaxial surfaces of all leaf samples.

Table S5. Similarity percentage analysis assessing the contribution of specific fungal genera to the dissimilarity between fungal communities on adaxial and abaxial surfaces of all leaf samples.

Table S6. List of core bacterial genera on adaxial and abaxial surfaces of leaves of tree species analyzed in this study.

Table S7. List of core fungal genera on adaxial and abaxial surfaces of leaves of tree species analyzed in this study.

Table S8. Bacterial metabolic pathways significantly more enriched on the adaxial leaf surfaces than on the abaxial surfaces.

Table S9. Bacterial metabolic pathways significantly more enriched on the abaxial leaf surfaces than on the adaxial surfaces.

Table S10. Metabolic pathways significantly enriched in the bacterial communities on the adaxial and abaxial leaf surfaces.

## Abbreviations

ASV: amplicon sequence variant
ITS2: internal transcribed spacer 2
JMA: Japan Meteorological Agency
LCC: largest connected component
LEfSe: Linear Discriminant Analysis Effect Size
NMDS: non-metric multidimensional scaling
PERMANOVA: permutational multivariate analysis of variance
PICRUSt2: Phylogenetic Investigation of Communities by Reconstruction of Unobserved States
TCA: tricarboxylic acid
