# Supplementary material for: Adaxial–abaxial leaf surface asymmetry is a key ecological driver of the phyllosphere microbiome
Source: J Exp Bot. 2026 May 29;77(17):5857–73. doi: 10.1093/jxb/erag255 (PMC13577749; doi:10.1093/jxb/erag255)
Supplement: Supplementary file 1 [file erag255_supplementary_data.zip › erag255 JEXBOT_2026_317787_Supplementary_Figures_Tables_revised_20260607.pdf]

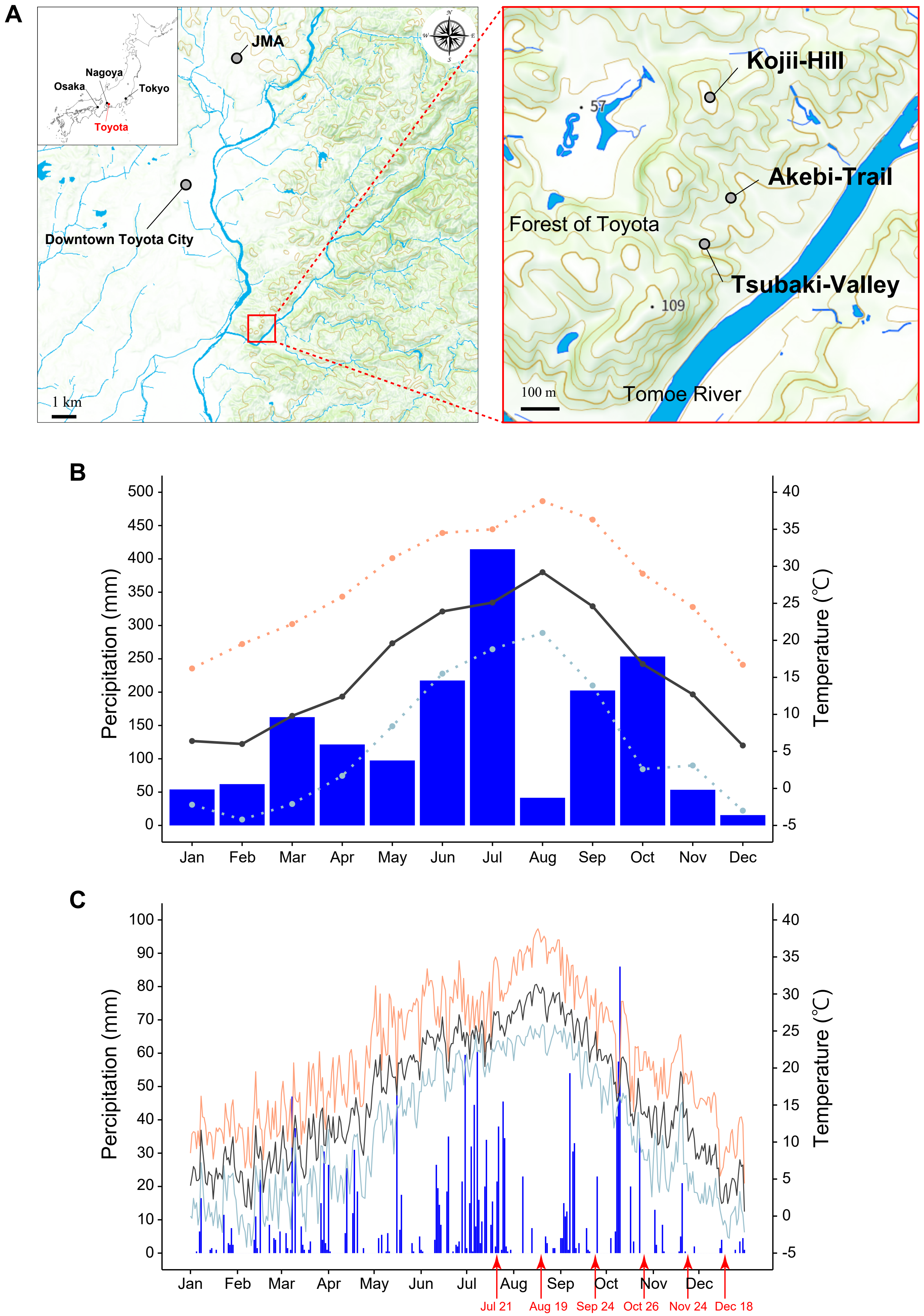
A
JMA
Nagoya
Osaka
Tokyo
Toyota
Downtown Toyota City
1 km
Kojii-Hill
57
Akebi-Trail
Forest of Toyota
Tsubaki-Valley
109
100 m
Tomoe River
B
Percipitation (mm)
Temperature (℃)
Jan
Feb
Mar
Apr
May
Jun
Jul
Aug
Sep
Oct
Nov
Dec
C
Percipitation (mm)
Temperature (℃)
Jul 21
Aug 19
Sep 24
Oct 26
Nov 24
Dec 18

Fig. S1. Sampling sites and climate conditions.

(A) Sampling sites. Topographic maps with 100 m (left) or 10 m (right) contour lines were obtained from the Geospatial Information Authority of Japan (https://www.gsi.go.jp/ENGLISH/index.html). The inset (top left) shows a broader area map. Three subsites in Forest of Toyota, located 6.25 km from downtown Toyota city, Aichi prefecture, Japan, are indicated: Tsubaki-Valley (35°2′11.4″N, 137°11′17.1″E; 57.8 m asl), Akebi-Trail (35°2′14.9″N, 137°11′19.5″E; 59.8 m asl), and Kojii-Hill (35°2′23.3″N, 137°11′17.5″E; 103.8 m asl).

(B) Monthly climate. Maximum (orange dotted line), average (black line), and minimum (light blue dotted line) temperatures, and monthly total precipitation (blue bar graphs) in 2020 were recorded at Toyota Meteorological Station, Japan Meteorological Agency (JMA; https://www.jma.go.jp/jma/indexe.html; 35°7.9′N, 137°10.6′E, 75 m asl), located 10.48 km from the subsites in Forest of Toyota. (C) Daily climate. Maximum (orange line), average (black line), and minimum (light blue line) temperatures, and daily precipitation (blue bar graphs) in 2020 were recorded at Toyota Meteorological Station, JMA. Red arrows show 6 dates (July 21, August 19, September 24, October 26, November 24, December 18), when leaf sampling was performed.

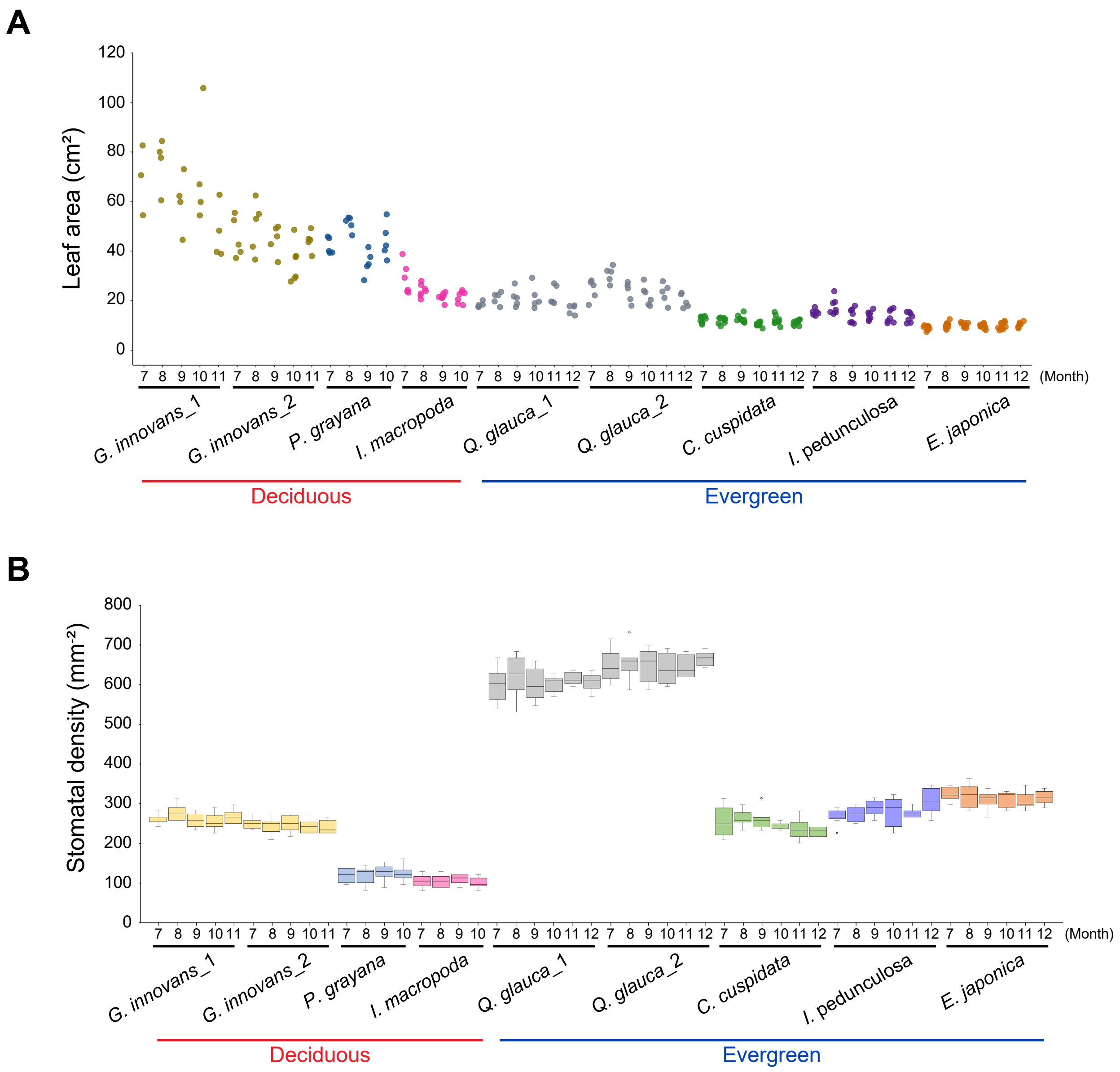

Fig. S2. Tree leaf (leaflet) parameters.

(A) Dot plot of leaf (or leaflet for *G. innovans*) area. Three to nine leaves (leaflets for *G. innovans*) were measured monthly from July to December 2020. (B) Box plot of stomatal density. Nine areas per leaf (three regions—tip, center, base—on each of three leaves (leaflets for *G. innovans*)) were measured monthly from July to December 2020. See Materials and Methods for details. Note that *G. innovans* lacks a December sample, and *P. grayana* and *I. macropoda* lack November and December samples since these are deciduous species.

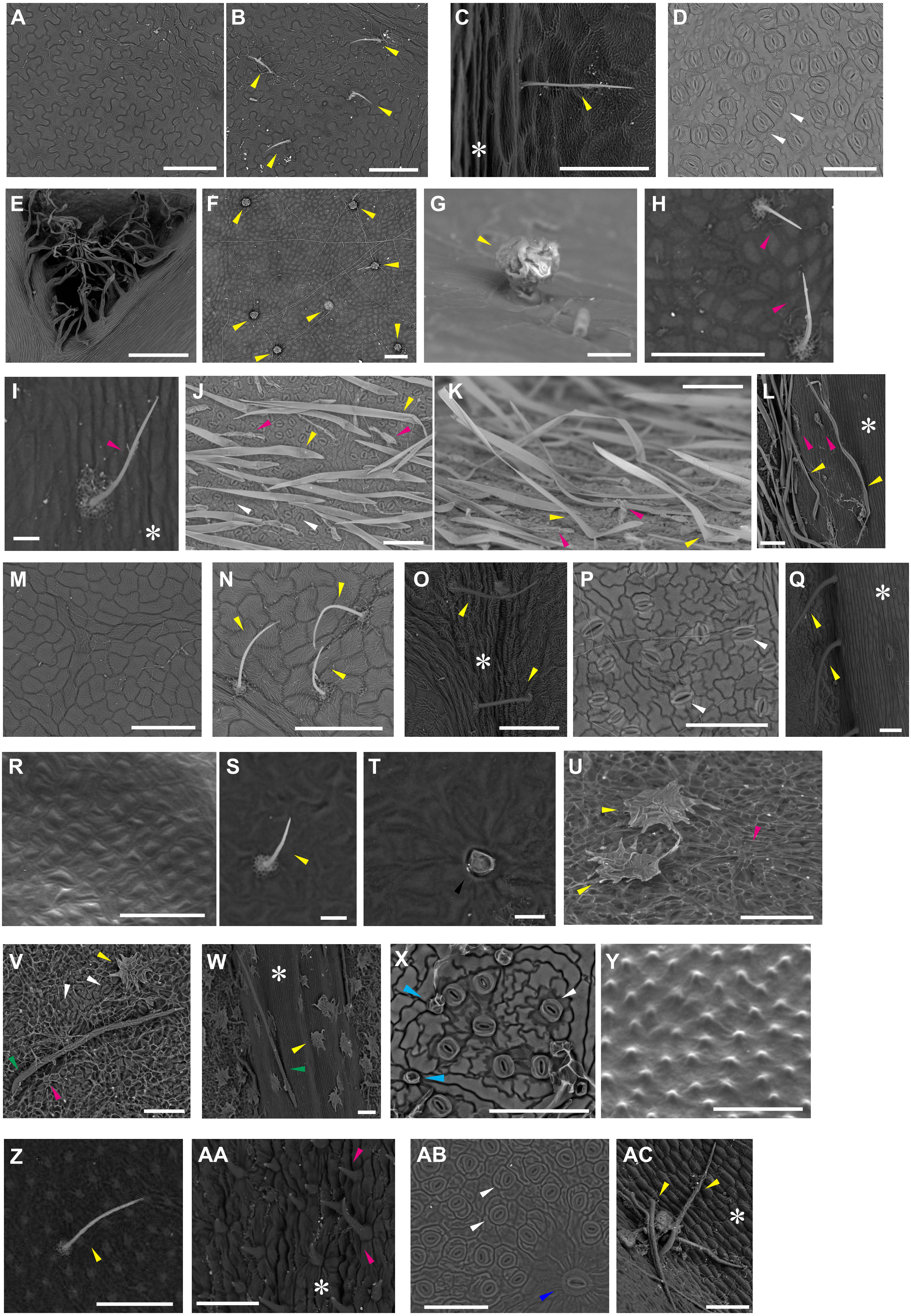
A
B
C
D
E
F
G
H
I
J
K
L
M
N
O
P
Q
R
S
T
U
V
W
X
Y
Z
AA
AB
AC

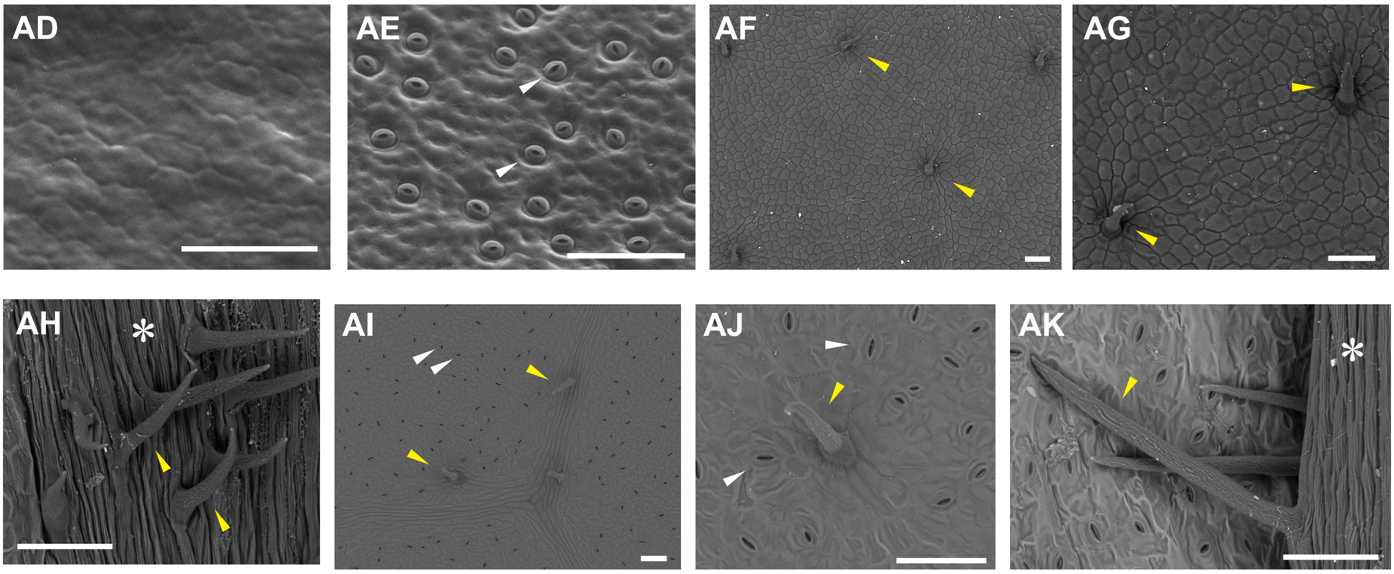

Fig. S3. Leaf epidermis of seven tree species under SEM.
See Supplementary Table S3 for detailed leaf configuration and vestiture. (A–C) Adaxial surface of *G. innovans*. Yellow arrowheads show uncinate solitary trichomes. Asterisk shows the primary vein. (D) Abaxial surface of *G. innovans*. White arrowheads show stomata; for clarity, only some are pointed. (E) The domatium, located in the axils between the primary and secondary veins of the abaxial surface of *G. innovans*. (F–I) Adaxial surfaces of *Q. glauca*. Yellow and magenta arrowheads show capitate glandular and uncinate solitary trichomes, respectively. The asterisk indicates the primary vein. (J–L) Abaxial surfaces of *Q. glauca*. Yellow, magenta, and white arrowheads show bifurcate trichomes, uniseriate solitary (pilate glandular) trichomes, and stomata, respectively; for clarity, only some are pointed. The asterisk indicates the primary vein. (M–O) Adaxial surfaces of *P. grayana*. Yellow arrowheads show uncinate solitary trichomes. The asterisk indicates the primary vein. (P, Q) Abaxial surfaces of *P. grayana*. Yellow and white arrowheads show uncinate solitary trichomes and stomata, respectively; for clarity, only some are pointed. The asterisk shows the primary vein. (R–T) Adaxial surfaces of *C. cuspidata*. The yellow arrowhead indicates an uncinate solitary trichome. A black arrowhead shows the trace left after a trichome was shed. (U–X) Abaxial surfaces of *C. cuspidata*. Magenta and yellow arrowheads indicate the short, scaly (boat-shaped) thin-walled peltate and the tall, shallow-lobed (rosulate) trichomes, respectively. Bluish green arrowheads indicate uncinate solitary trichomes. White arrowheads show stomata. For clarity, only some are pointed. The asterisk indicates the primary vein. Image X shows the abaxial leaf surface after the trichomes have been peeled off with adhesive tape. Sky blue arrowheads indicate the trichome bases. (Y–AA) Adaxial surfaces of *I. pedunculosa*. Yellow and magenta arrowheads indicate thin and thicker uncinate solitary trichomes, respectively; for clarity, only some are pointed. The asterisk indicates the primary vein. (AB, AC) Abaxial surfaces of *I. pedunculosa*. Yellow and white arrowheads indicate uncinate solitary trichomes and stomata, respectively; for clarity, only some are pointed. The blue arrowhead indicates a giant stoma. The asterisk indicates the primary vein. (AD) Adaxial surfaces of *E. japonica*. (AE) Abaxial surfaces of *E. japonica*. White arrowheads indicate stomata; for clarity, only some are pointed. (AF–AH) Adaxial surfaces of *I. macropoda*. Yellow arrowheads indicate uncinate solitary trichomes; for clarity, only some are pointed. The asterisk indicates the primary vein. (AI–AK) Abaxial surfaces of *I. macropoda*. Yellow and white arrowheads indicate uncinate solitary trichomes and stomata, respectively; for clarity, only some are pointed. The asterisk indicates the primary vein. Images R, Y, AD, AE were taken with an SU3500 scanning electron microscope (Hitachi), and all other images were taken with a TM3000 scanning electron microscope (Hitachi). Images C, E, H, I, N, O, R–T, Y–AA, AD, AE, AH were taken after light swabbing because of the presence of excessive dust and/or microorganisms covering the surface; all other images were taken without any treatment (as sampled in the field). Scale bars, 20 µm (G, I, S, T); 100 µm (A–D, F, H, J–R, U–Z, AA–AK); 500 µm (E).

A

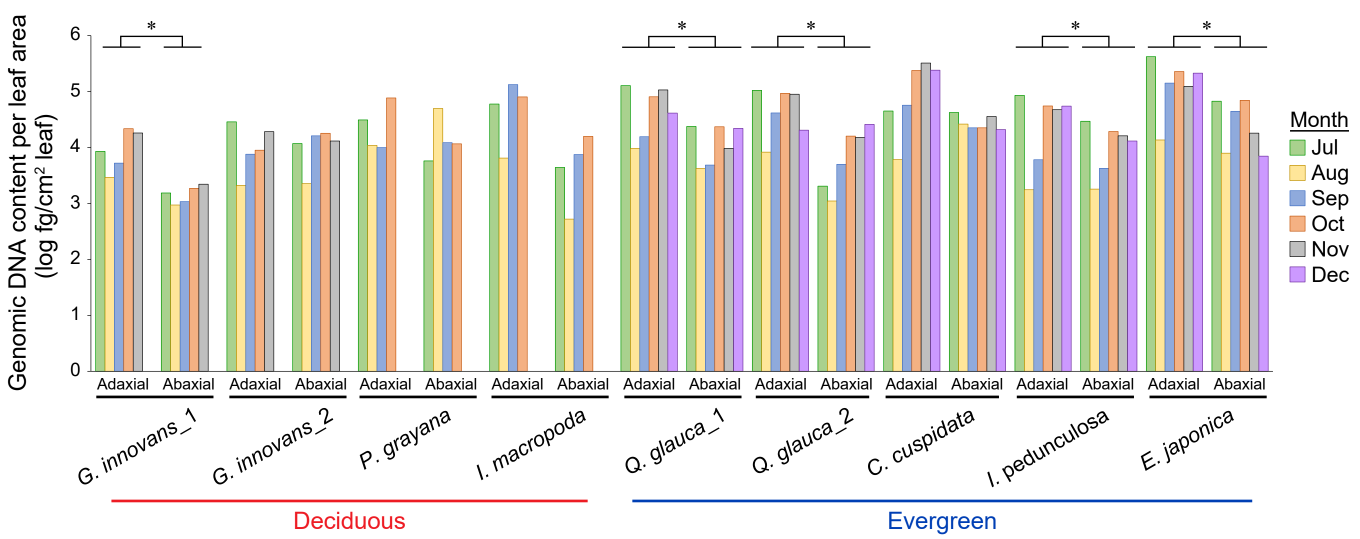

B

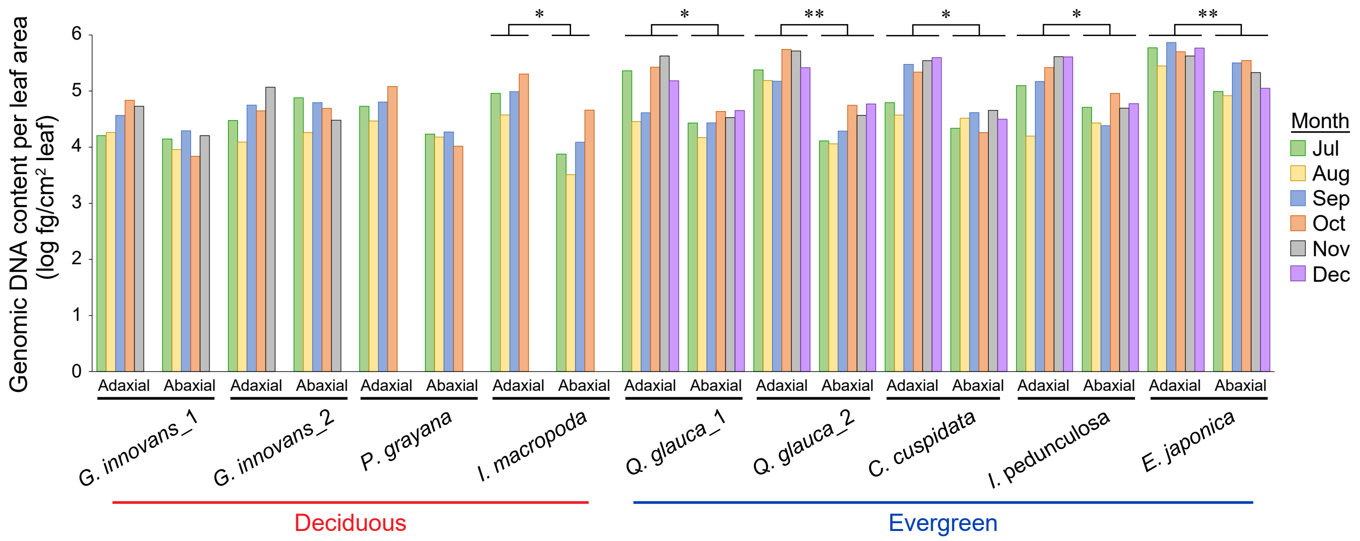

Fig. S4. Microbial genomic DNA content on the adaxial and abaxial surfaces of tree leaves.
The amount of bacterial (A) and fungal (B) DNA per adaxial and abaxial surfaces of tree leaves. Bacterial and fungal DNA content was estimated through quantitative real-time PCR targeting 16S rRNA and 28S rRNA, respectively, using a microbial community DNA standard (Zymo Research, Irvine, CA), and then divided by the leaf area swabbed for DNA extraction. Note that *G. innovans* lacks the December sample, and *P. grayana* and *I. macropoda* lack November and December samples since these trees are deciduous species. Values represent the mean of three technical replicates. *, $P < 0.05$; **, $P < 0.01$ (two-sided paired t-test).

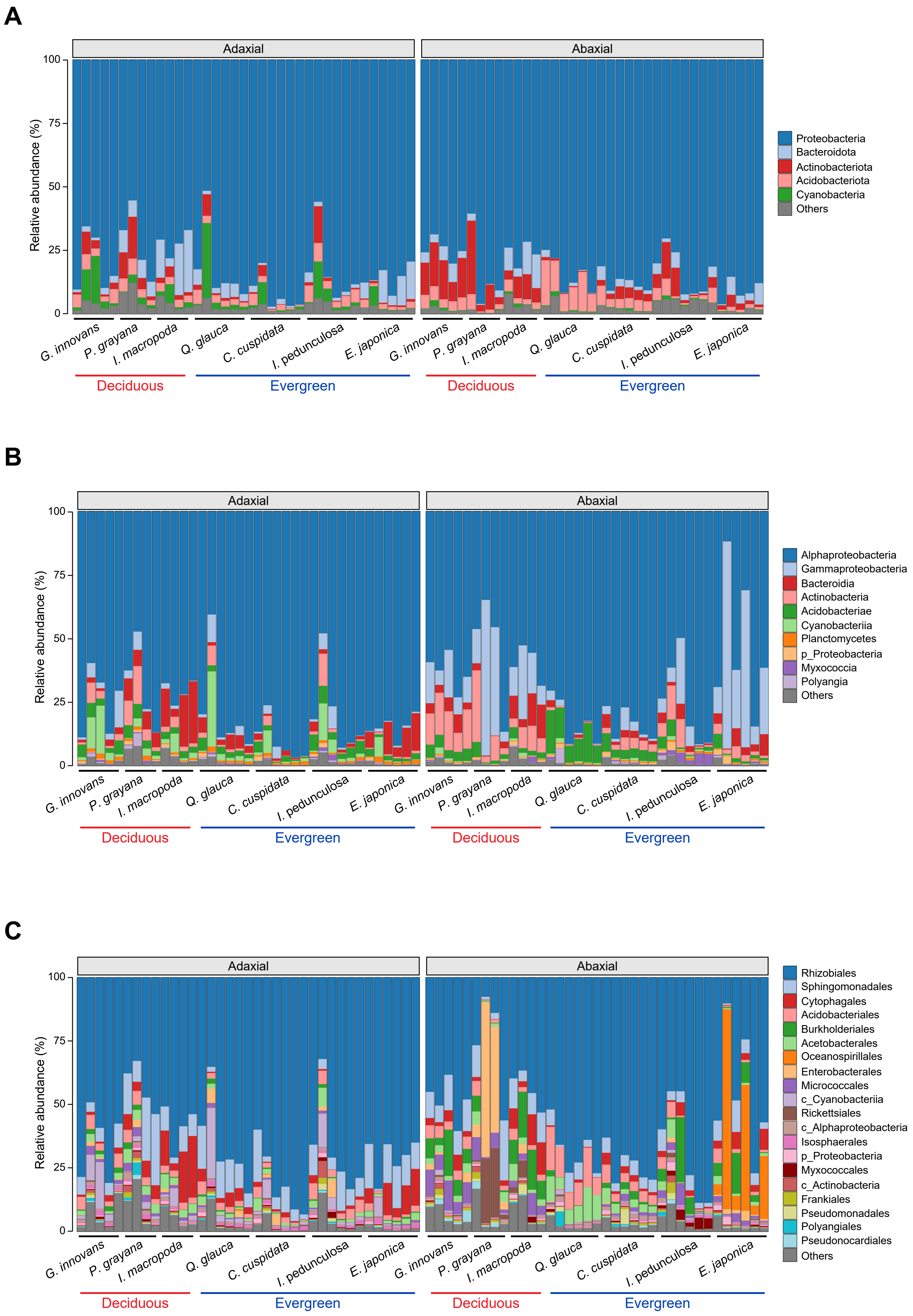

A
B
C
Adaxial
Abaxial
Relative abundance (%)
100
75
50
25
0
G. innovans
P. grayana
I. macropoda
Q. glauca
C. cuspidata
I. pedunculosa
E. japonica
Deciduous
Evergreen
Proteobacteria
Bacteroidota
Actinobacteriota
Acidobacteriota
Cyanobacteria
Others
Alphaproteobacteria
Gammaproteobacteria
Bacteroidia
Actinobacteria
Acidobacteriae
Cyanobacteriia
Planctomycetes
p_Proteobacteria
Myxococcia
Polyangia
Others
Rhizobiales
Sphingomonadales
Cytophagales
Acidobacteriales
Burkholderiales
Acetobacterales
Oceanospirillales
Enterobacterales
Micrococcales
c_Cyanobacteriia
Rickettsiales
c_Alphaproteobacteria
Isosphaerales
p_Proteobacteria
Myxococcales
c_Actinobacteria
Frankiales
Pseudomonadales
Polyangiales
Pseudonocardiales
Others

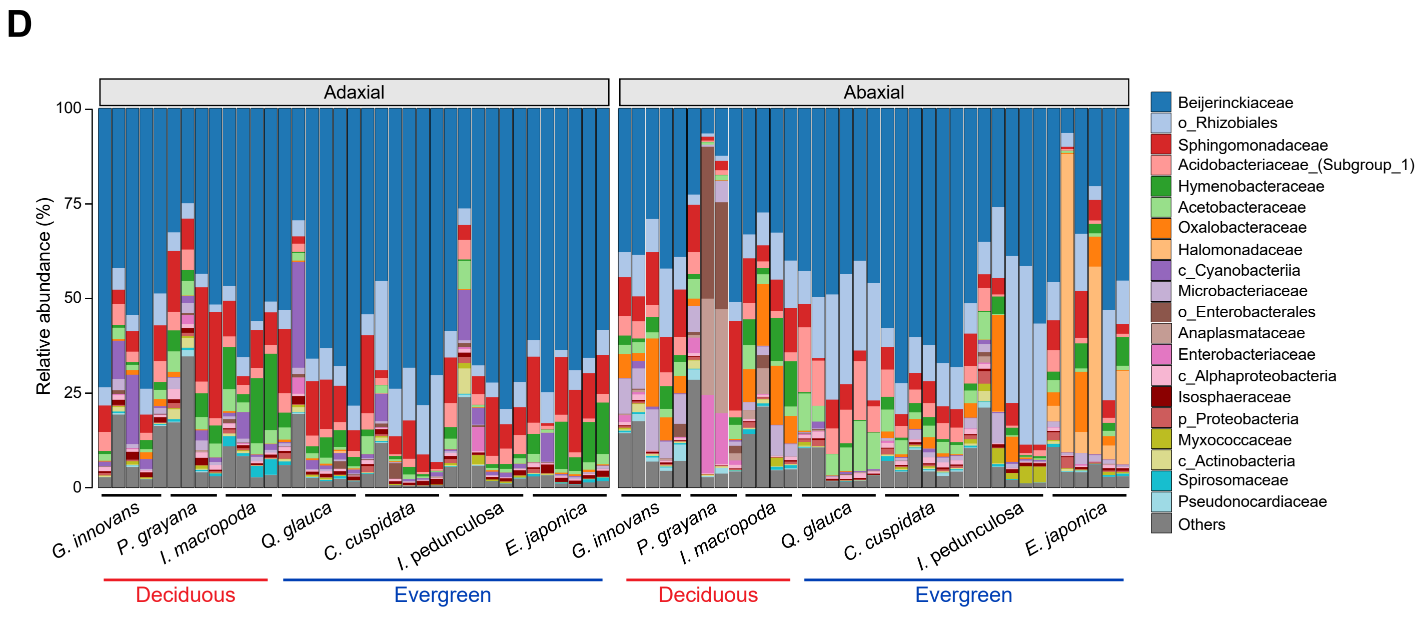

Fig. S5. Bacterial community compositions.

Relative abundance at (A) phylum (top 5), (B) class (top 10), (C) order (top 20), (D) family (top 20) levels. The order in each sample is July, August, September, October, November, and December. Samples for *G. innovans* could not be obtained in December, and those for *P. grayana* and *I. macropoda* were unavailable in November and December since these trees are deciduous species.

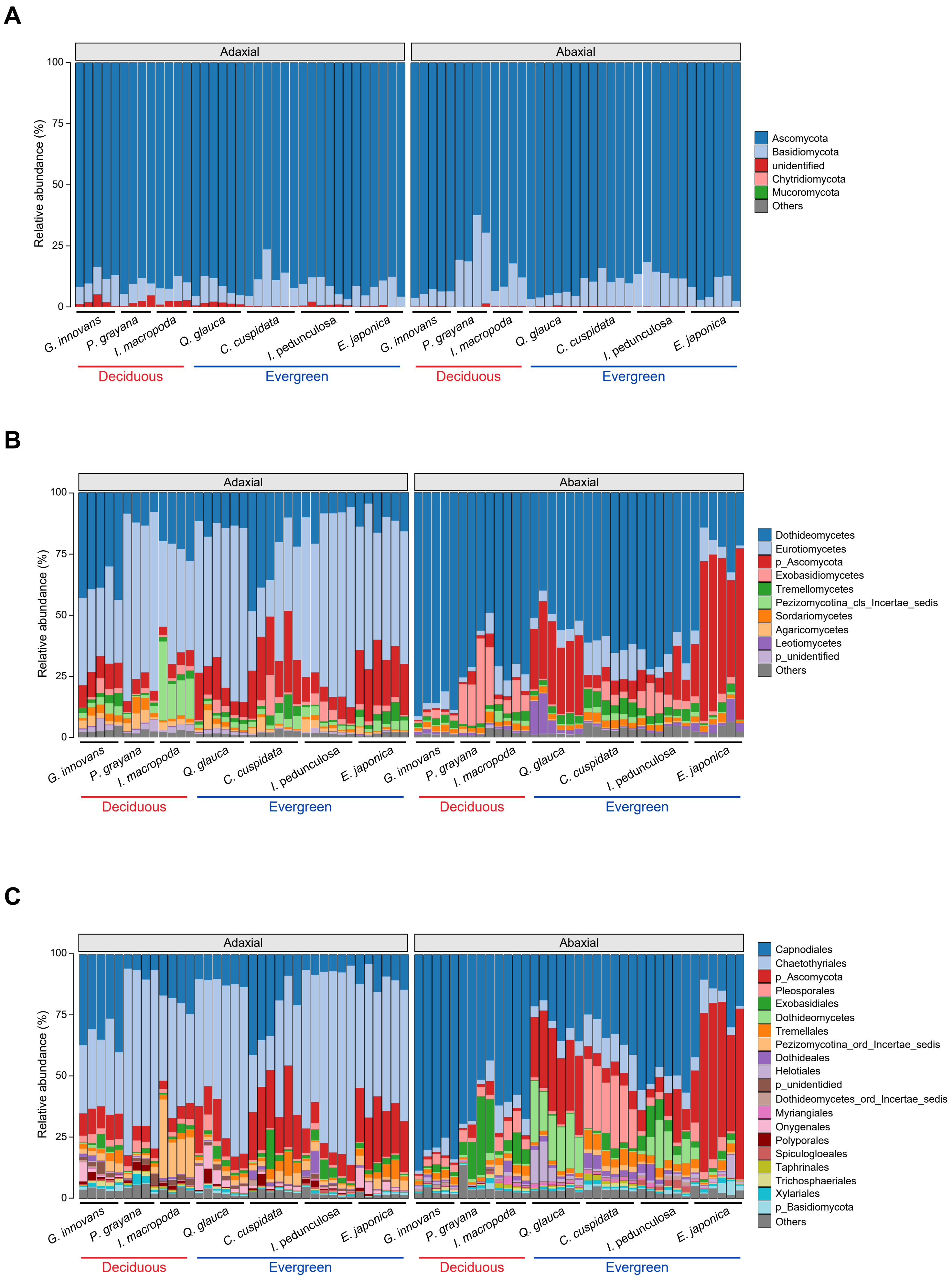
A
Adaxial
Abaxial
Relative abundance (%)
100
75
50
25
0
G. innovans
P. grayana
I. macropoda
Q. glauca
C. cuspidata
I. pedunculosa
E. japonica
Deciduous
Evergreen
Ascomycota
Basidiomycota
unidentified
Chytridiomycota
Mucoromycota
Others
B
Dothideomycetes
Eurotiomycetes
p_Ascomycota
Exobasidiomycetes
Tremellomycetes
Pezizomycotina_cls_Incertae_sedis
Sordariomycetes
Agaricomycetes
Leotiomycetes
p_unidentified
Others
C
Capnodiales
Chaetothyriales
p_Ascomycota
Pleosporales
Exobasidiales
Dothideomycetes
Tremellales
Pezizomycotina_ord_Incertae_sedis
Dothideales
Helotiales
p_unidentified
Dothideomycetes_ord_Incertae_sedis
Myriangiales
Onygenales
Polyporales
Spiculogloeales
Taphrinales
Trichosphaeriales
Xylariales
p_Basidiomycota
Others

D

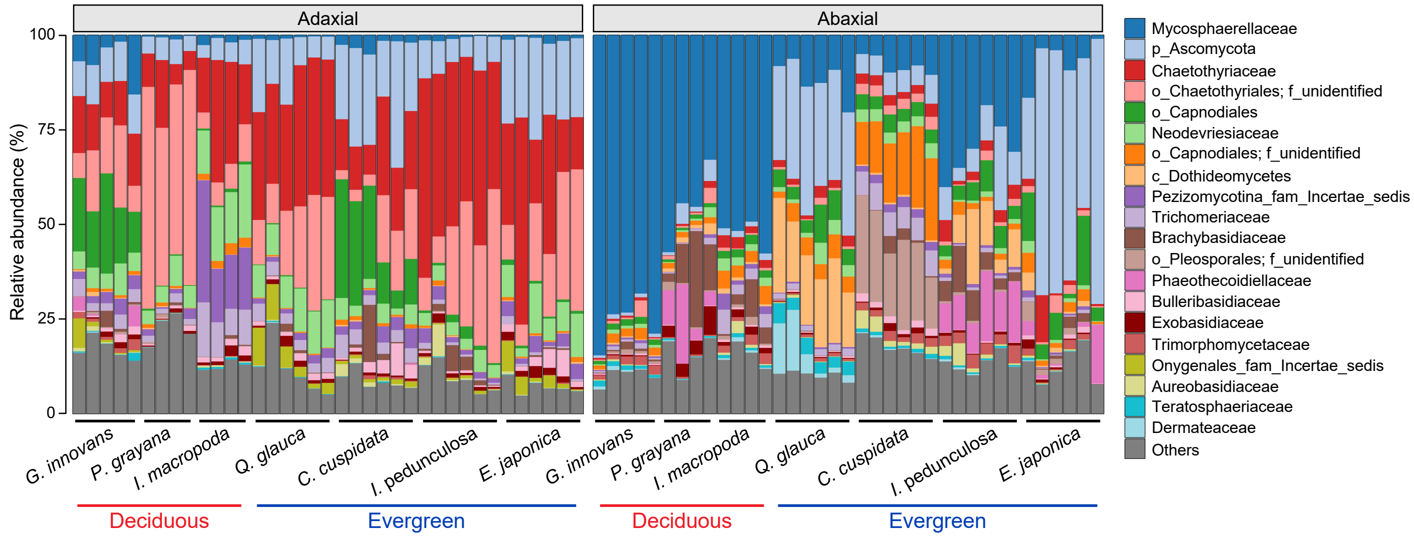

Fig. S6. Fungal community compositions.
Relative abundance at (A) phylum (top 5), (B) class (top 10), (C) order (top 20), (D) family (top 20) levels. The order in each sample is July, August, September, October, November, and December. Samples for *G. innovans* could not be obtained in December, and those for *P. grayana* and *I. macropoda* were unavailable in November and December since these trees are deciduous species.

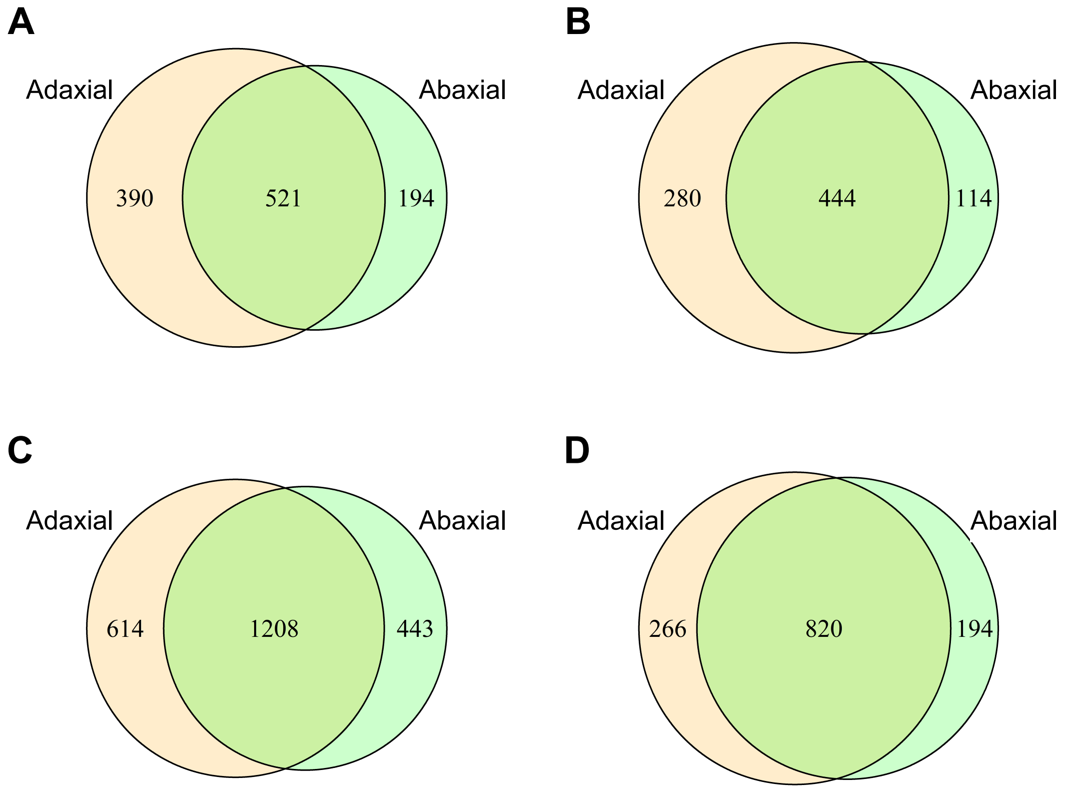

Fig. S7. Venn diagrams of unique and shared microbial taxa between adaxial and abaxial leaf compartments, shown at the lowest taxonomic and genus levels.
(A, B) Bacterial communities. (C, D) Fungal communities. (A, C) Lowest taxonomic level. (B, D) Genus level.

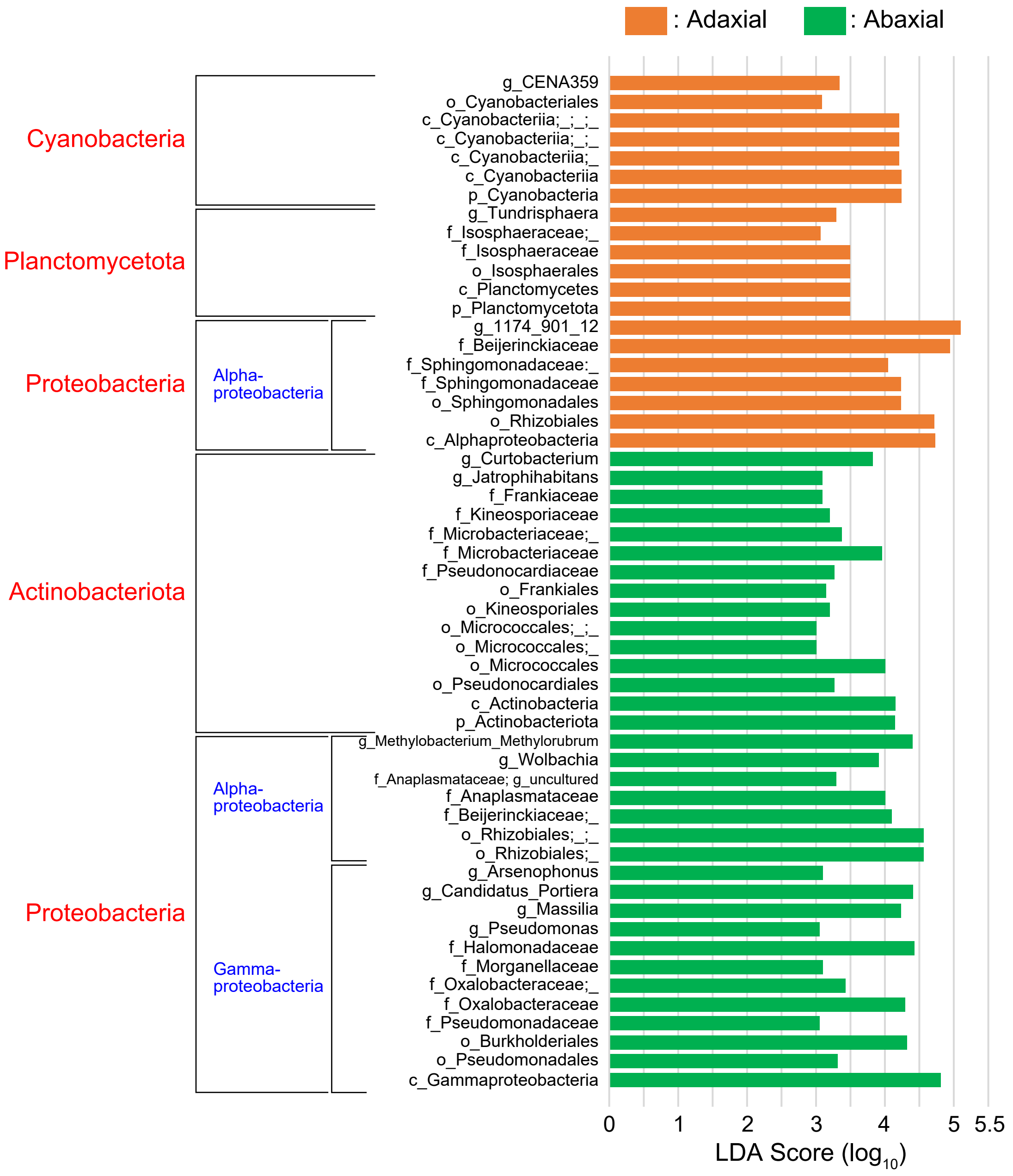

Fig. S8. Differential abundance analysis of bacterial taxa between adaxial and abaxial sides of leaf surfaces.

Linear discriminant analysis (LDA) effect size (LEfSe) analysis identified differentially abundant bacterial taxa between adaxial and abaxial leaf surfaces (LDA scores >3.0).

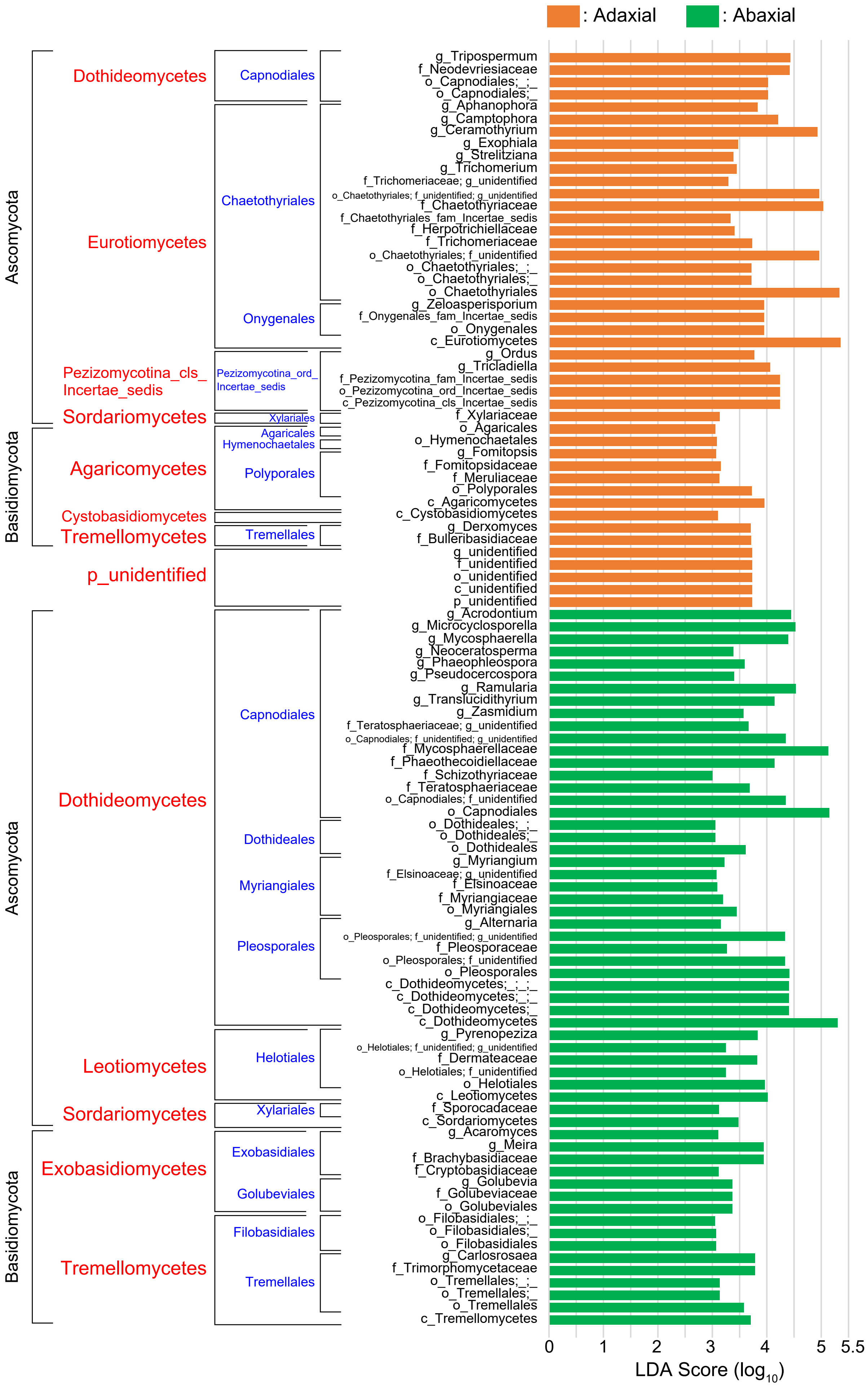

Fig. S9. Differential abundance analysis of fungal taxa between adaxial and abaxial sides of leaf surfaces. Linear discriminant analysis (LDA) effect size (LEfSe) analysis identified differentially abundant fungal taxa between adaxial and abaxial leaf surfaces (LDA scores >3.0).

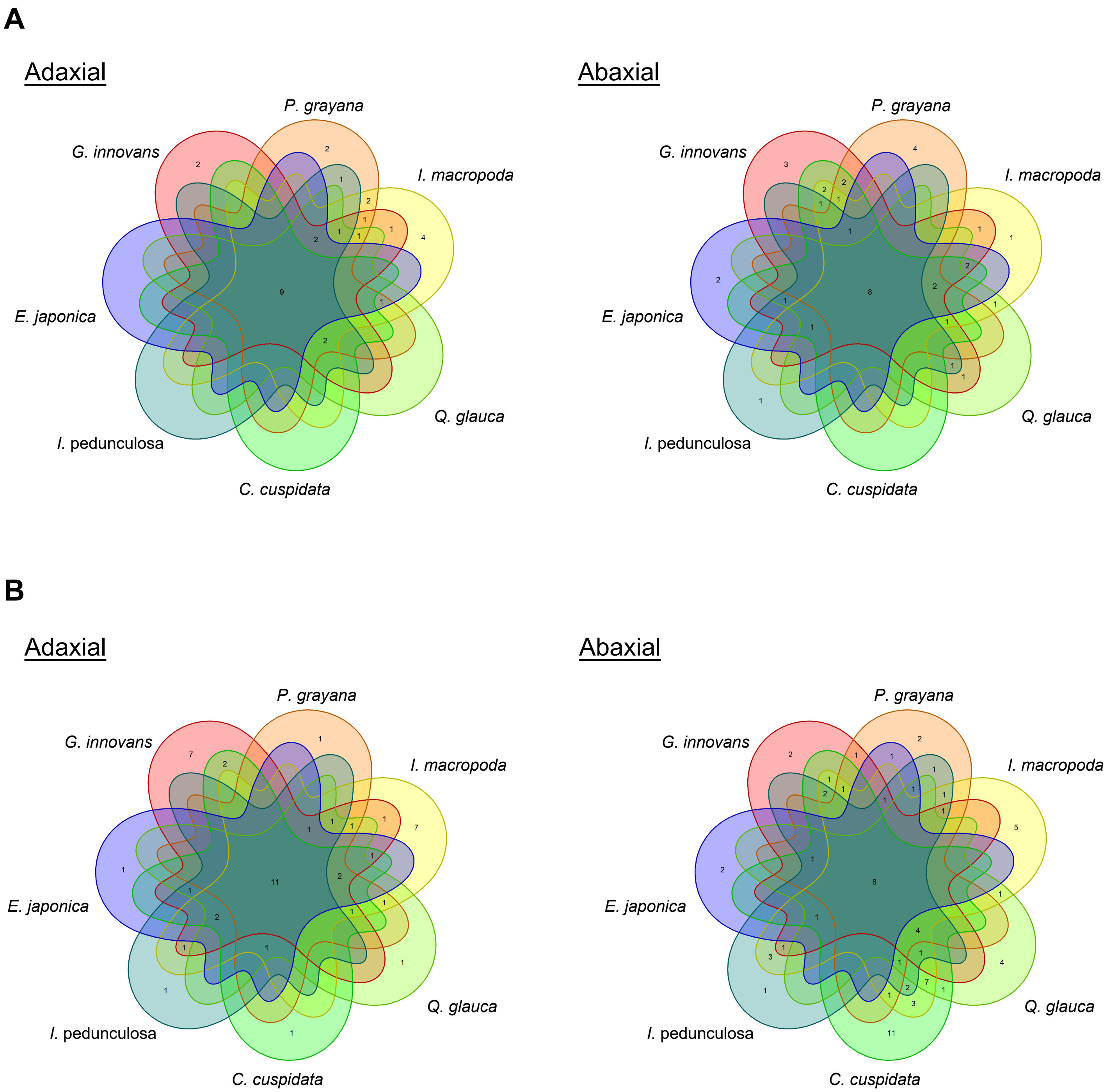

Fig. S10. Venn diagrams of microbial genera by host species.
Bacterial and fungal genera present consistently (>0.1% relative abundance) across all time points were counted. Zero values were excluded for clarity. (A) Bacterial genera; (B) Fungal genera.

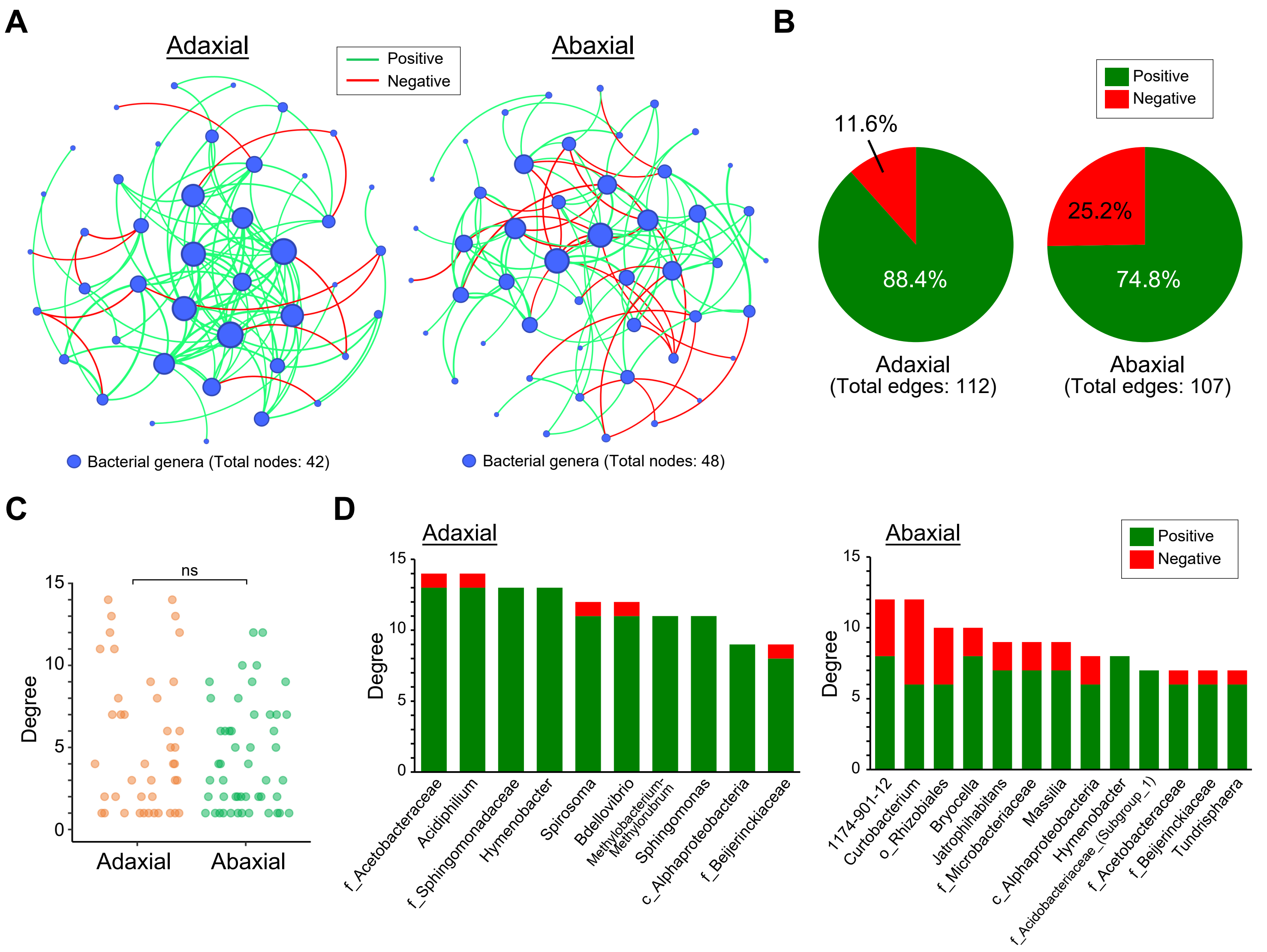

Fig. S11. Bacterial intra-kingdom co-occurrence networks on leaf surfaces.

(A) Bacterial co-occurrence networks on adaxial and abaxial surfaces of all trees analyzed. Blue nodes represent bacterial genera. The node size depends on the degree of connection. The edge color shows positive (light green) and negative (red) correlations. (B) Proportion of positive and negative correlations in bacterial networks on adaxial and abaxial surfaces. (C) Degree values of bacterial genera in co-occurrence networks on leaf surfaces (ns, not significant; Wilcoxon rank sum test). (D) Degree and correlation type of the top 10 nodes in bacterial networks.

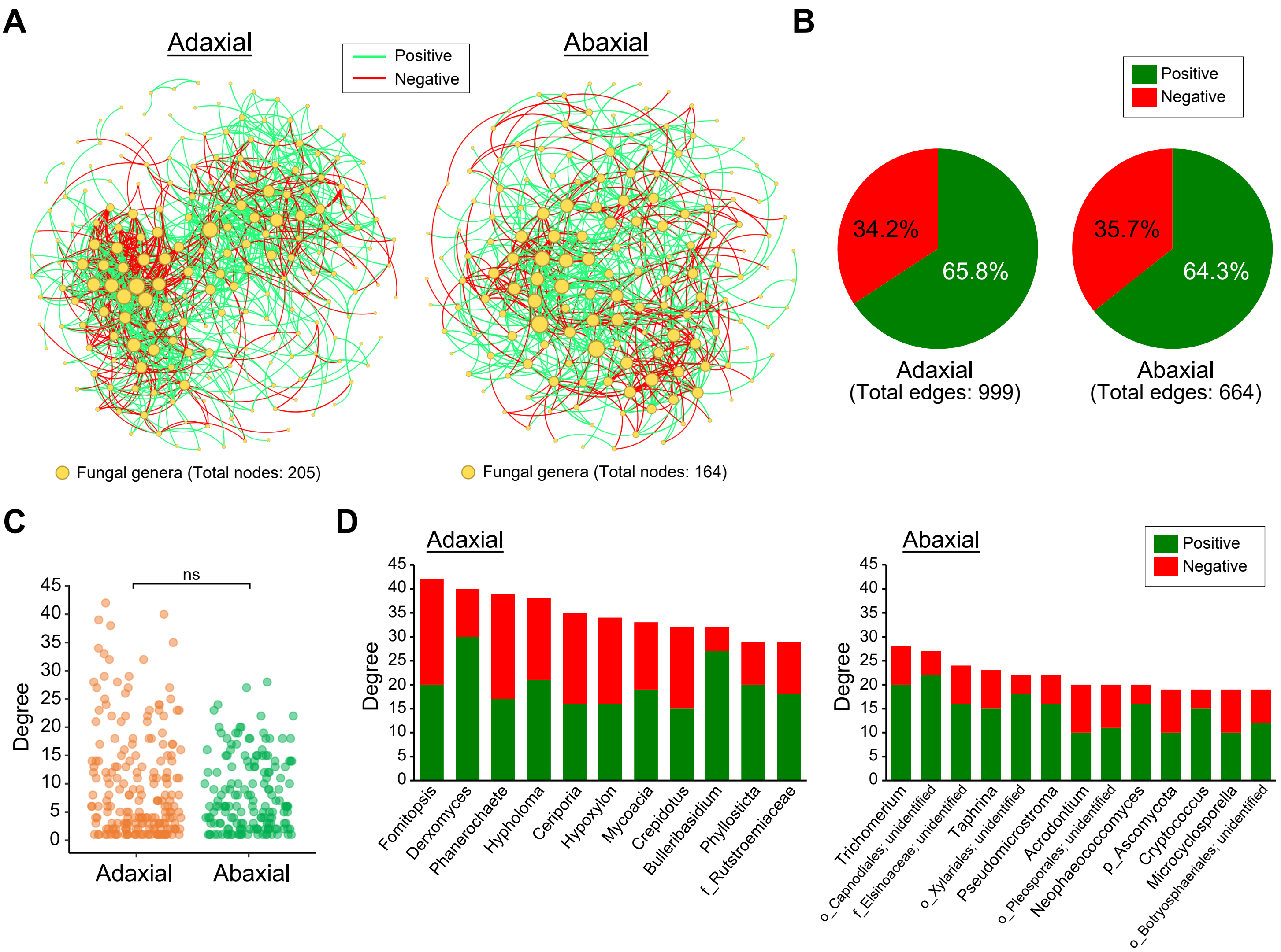

Fig. S12. Fungal intra-kingdom co-occurrence networks on leaf surfaces.

(A) Fungal co-occurrence networks on adaxial and abaxial surfaces of all trees analyzed. Yellow nodes represent fungal genera. The node size depends on the degree of connection. The edge color shows positive (light green) and negative (red) correlations. (B) Proportion of positive and negative correlations in fungal networks on adaxial and abaxial surfaces. (C) Degree values of fungal genera in co-occurrence networks on leaf surfaces (ns, not significant; Wilcoxon rank sum test). (D) Degree and correlation type of the top 10 nodes in fungal networks.

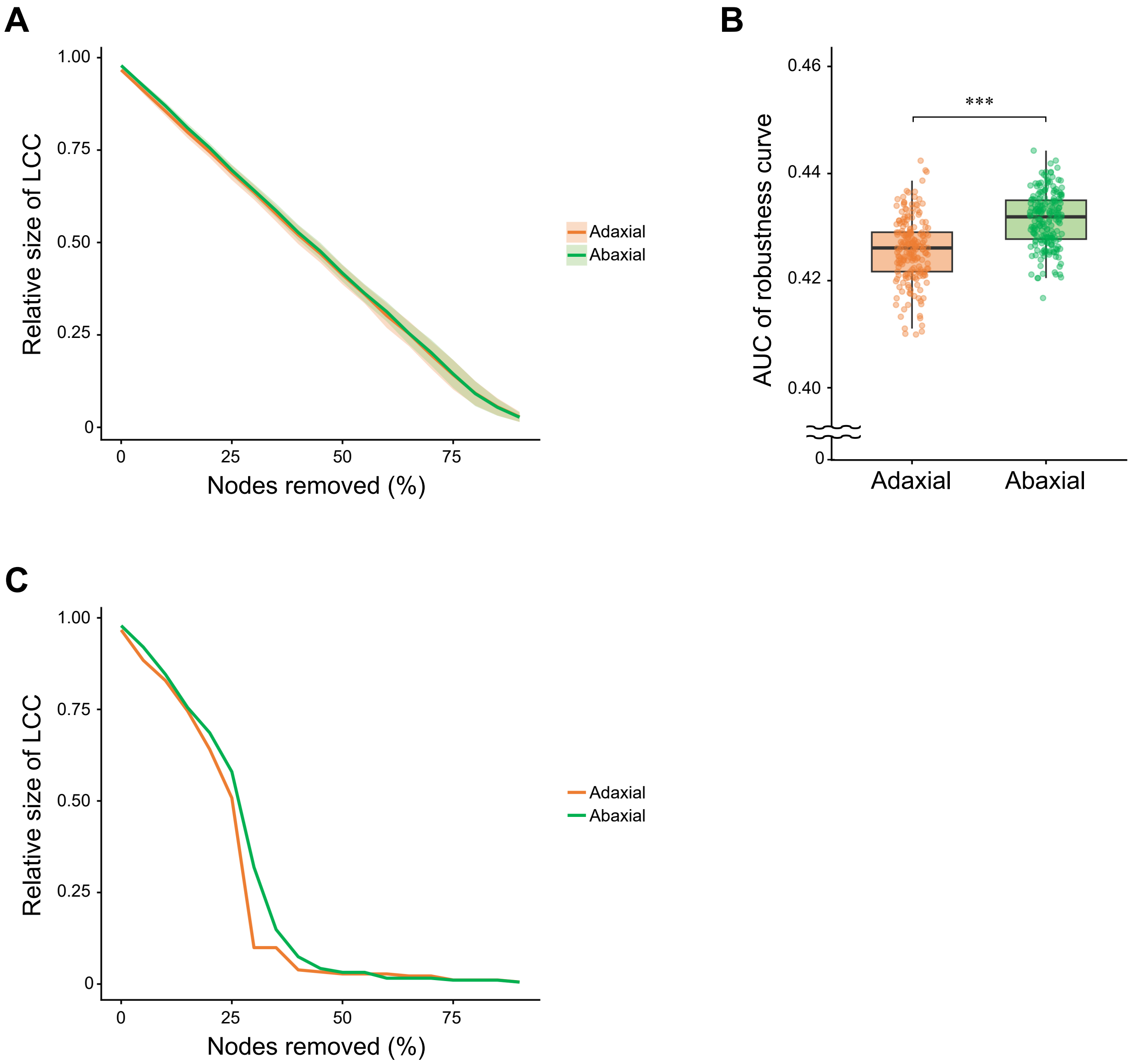

Fig. S13. Robustness analysis of inter-kingdom microbial interaction networks on adaxial and abaxial leaf surfaces.

(A) Random node removal analysis. Robustness of the microbial interaction networks is evaluated using random node removal simulations. Nodes are removed randomly at increasing fractions of the total network nodes (0–90%), and the relative size of the largest connected component (LCC) is measured after each removal step. Lines represent the mean relative LCC across 200 random-removal simulations, and shaded areas indicate ± SD. (B) Comparison of robustness between adaxial and abaxial microbial networks. Robustness under random node removal is assessed using the area under the LCC–removal curve (AUC) calculated for each simulation replicate. Boxplots represent the distribution of AUC values across simulations, with individual points indicating replicate values. ***, $P < 0.001$; Wilcoxon rank-sum test. (C) Targeted node removal analysis. Robustness of the microbial interaction networks is evaluated based on node degree; whereby high-degree nodes are removed in descending order of their initial degree, representing the loss of highly connected taxa. The relative size of the LCC is scored after each removal step.

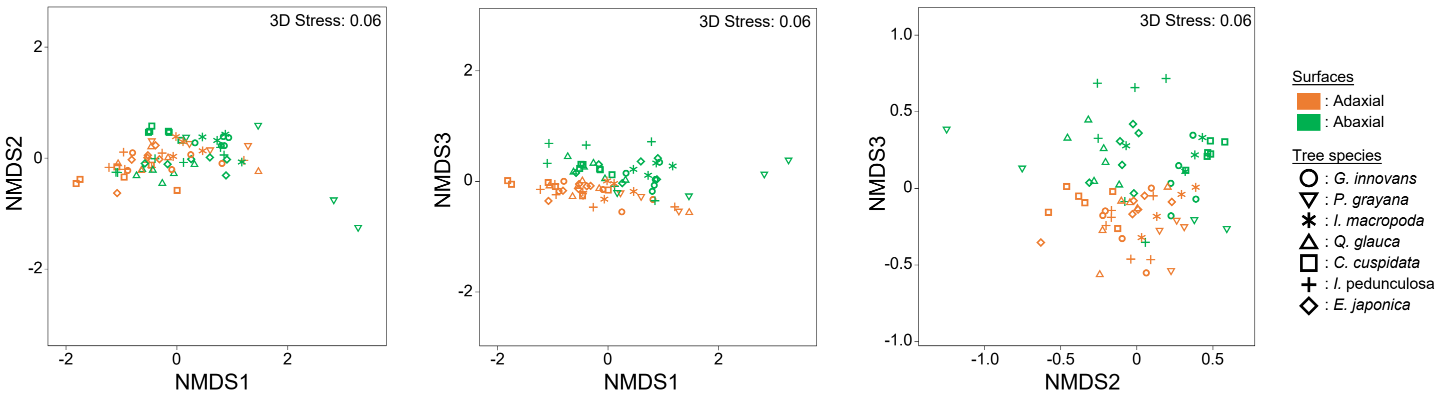

Fig. S14. Non-metric multidimensional scaling ordination in three dimensions using Bray–Curtis dissimilarity of the predicted metabolic pathways of the bacterial communities on adaxial and abaxial sides of leaf surfaces.

(*Left*) First and second axes. (*Center*) First and third axes. (*Right*) Second and third axes. Each point corresponds to a sample, with the different colors representing leaf surfaces, and the different shapes representing tree species.

Table S1. Tree species characteristics and sampling design.

Scientific Name	Order	Family	Leaf Habitat	No. of individuals	Sampling sites	Months sampled
Gamblea innovans	*Apiales*	*Araliaceae*	Deciduous	2	Tsubaki-Valley, Kojii-Hill	Jul-Nov
Prunus grayana	*Rosales*	*Rosaceae*	Deciduous	1	Akebi-Trail	Jul-Oct
Ilex macropoda	*Aquifoliales*	*Aquifoliaceae*	Deciduous	1	Kojii-Hill	Jul-Oct
Quercus glauca	*Fagales*	*Fagaceae*	Evergreen	2	Tsubaki-Valley, Kojii-Hill	Jul-Dec
Castanopsis cuspidata	*Fagales*	*Fagaceae*	Evergreen	1	Kojii-Hill	Jul-Dec
Ilex pedunculosa	*Aquifoliales*	*Aquifoliaceae*	Evergreen	1	Kojii-Hill	Jul-Dec
Eurya japonica	*Ericales*	*Pentaphylacaceae*	Evergreen	1	Kojii-Hill	Jul-Dec

Table S2. Primers for quantitative real-time PCR and amplicon sequencing.

Target		Primer sequences (5' to 3')	References
For quantitative real-time PCR			
Bacterial 16S rRNA	Forward	AGRGTTTGATYMTGGCTCAG	Toyoda *et al*., 2023
	Reverse	TGCTGCCTCCCGTAGGAGT	
Fungal 28S rRNA	Forward	GTGAAATTGTTGAAAGGGAA	Sandhu *et al*., 1995
	Reverse	GACTCCTTGGTCCGTGTT	
For amplicon sequencing			
Bacterial 16S rRNA (V1-V2)	Forward	TCGTCGGCAGCGTCAGATGTGTATAAGAGACAGAGRG<br>TTTGATYMTGGCTCAG	Kim *et al*., 2013
	Reverse	GTCTCGTGGGCTCGGAGATGTGTATAAGAGACAGTGC<br>TGCCTCCCGTAGGAGT	
Fungal ITS2	Forward	TCGTCGGCAGCGTCAGATGTGTATAAGAGACAGGCAT<br>CGATGAAGAACGCAGC	Bellemain *et al*., 2010
	Reverse	GTCTCGTGGGCTCGGAGATGTGTATAAGAGACAGTCC<br>TCCGCTTATTGATATGC	

Underlining regions indicate Illumina overhang adapter sequences included in the amplicon sequencing primers.

Table S4. Similarity percentage analysis assessing the contribution of specific bacterial genera to the dissimilarity between bacterial communities on adaxial and abaxial surfaces of all leaf samples.

Average dissimilarity between leaf surfaces = 51.32	Av.Abund					
Bacterial genera	Adaxial	Abaxial	Av.Diss	Diss/SD	Contrib%	Cum.%
1174-901-12	**11451.58**	5804.50	13.57	1.54	26.45	26.45
o_*Rhizobiales*;_;_	1485.75	**3355.17**	5.17	0.86	10.07	36.52
Methylobacterium-Methylorubrum	1384.50	**2365.40**	3.98	1.13	7.75	44.27
f_*Beijerinckiaceae*;_	723.38	**1346.33**	2.00	1.18	3.90	48.17
c_*Cyanobacteriia*;_;_;_	**911.35**	99.40	1.90	0.44	3.70	51.88
Hymenobacter	**857.50**	526.19	1.89	0.89	3.69	55.56
f_*Sphingomonadaceae*;_	**1213.58**	830.52	1.88	1.23	3.66	59.22
Massilia	97.98	**869.46**	1.84	0.67	3.59	62.81
Candidatus_Portiera	0.31	**797.69**	1.74	0.26	3.38	66.20
Sphingomonas	**956.60**	693.94	1.55	0.90	3.03	69.23
f_*Acetobacteraceae*;_	282.75	**675.65**	1.27	0.58	2.48	71.71

The analysis was conducted based on the Bray–Curtis similarity index using a one-way design comparing adaxial and abaxial leaf surfaces. Bacterial genera responsible for >70% of the dissimilarity between adaxial and abaxial surfaces are listed.
Av. Abund: average abundance; Av. Diss: average dissimilarity; Diss/SD: dissimilarity divided by standard deviation; Contrib%: % contribution to the within-group dissimilarity; Cum. %: cumulative % contribution to the within-group dissimilarity. The values for the surface with the higher average abundance are indicated in bold.

Table S5. Similarity percentage analysis assessing the contribution of specific fungal genera to the dissimilarity between fungal communities on adaxial and abaxial surfaces of all leaf samples.

Average dissimilarity between leaf surfaces = 77.99	Av.Abund					
Fungal genera	Adaxial	Abaxial	Av.Diss	Diss/SD	Contrib%	Cum.%
o_*Chaetothyriales*; unidentified; unidentified	**13919.40**	967.85	9.03	1.12	11.58	11.58
Ceramothyrium	**12719.60**	543.92	8.49	1.15	10.88	22.46
p_*Ascomycota*;_;_;_;_	8810.13	**11956.63**	7.91	1.04	10.15	32.61
Ramularia	8.23	**5452.73**	3.79	0.64	4.85	37.47
Microcyclosporella	479.79	**5466.06**	3.71	0.89	4.76	42.23
o_*Capnodiales*;_;_	**4547.58**	2901.54	3.52	0.88	4.51	46.74
Acrodontium	137.29	**4903.02**	3.33	0.76	4.27	51.00
Mycosphaerella	718.48	**5163.77**	3.29	0.83	4.21	55.22
c_*Dothideomycetes*;_;_;_	140.19	**4567.48**	3.10	0.69	3.97	59.19
Tripospermum	**5165.06**	1134.17	2.89	1.28	3.71	62.90
o_*Capnodiales*; unidentified; unidentified	489.94	**3289.31**	1.99	0.77	2.55	65.45
Camptophora	**3298.56**	834.40	1.93	0.60	2.48	67.93
o_*Pleosporales*; unidentified; unidentified	130.60	**2289.69**	1.54	0.45	1.98	69.91
Meira	807.42	**1893.52**	1.48	0.64	1.90	71.81

The analysis was conducted based on the Bray–Curtis similarity index using a one-way design comparing adaxial and abaxial leaf surfaces. Fungal genera responsible for >70% of the dissimilarity between adaxial and abaxial surfaces are listed.
Av. Abund: average abundance; Av. Diss: average dissimilarity; Diss/SD: dissimilarity divided by standard deviation; Contrib%: % contribution to the within-group dissimilarity; Cum. %: cumulative % contribution to the within-group dissimilarity. The values for the surface with the higher average abundance are indicated in bold.

Table S6. List of core bacterial genera on adaxial and abaxial surfaces of leaves of tree species analyzed in this study.

Leaf surface	Bacterial genera	Leaf surface	Bacterial genera
Adaxial	*1174-901-12*	Abaxial	*1174-901-12*
	Bryocella		*Methylobacterium-Methylorubrum*
	Methylobacterium-Methylorubrum		*Sphingomonas*
	Sphingomonas		f_*Acetobacteraceae*;_
	Tundrisphaera		f_*Beijerinckiaceae*;_
	f_*Acetobacteraceae*;_		f_*Sphingomonadaceae*;_
	f_*Beijerinckiaceae*;_		o_*Rhizobiales*;_;_
	f_*Sphingomonadaceae*;_		c_*Alphaproteobacteria*;_;_;_
	o_*Rhizobiales*;_;_		

Table S7. List of core fungal genera on adaxial and abaxial surfaces of leaves of tree species analyzed in this study.

Leaf surface	Fungal genera	Leaf surface	Fungal genera
Adaxial	*Camptophora*	Abaxial	*Acrodontium*
	Ceramothyrium		*Mycosphaerella*
	Derxomyces		*Tripospermum*
	Exophiala		o_*Capnodiales*;_;_
	Ordus		o_*Capnodiales*; unidentified;_
	Trichomerium		o_*Chaetothyriales*; unidentified
	Tripospermum		c_*Dothideomycetes*;_;_;_
	o_*Capnodiales*;_;_		p_*Ascomycota*;_;_;_;_
	o_*Chaetothyriales*; unidentified;_		
	p_*Ascomycota*;_;_;_;_		
	p_*Basidiomycota*;_;_;_;_		

Table S10. Metabolic pathways significantly enriched in the bacterial communities on the adaxial and abaxial leaf surfaces.

Metabolic (MetaCyc) pathways (Class categories)	No. of pathways (%)[a]	
	Adaxial surface	Abaxial surface
**Superclasses:** Activation/Inactivation/Interconversion		
Interconversions	0 (0.0)	1 (1.1)
**Superclasses:** Biosynthesis		
Amine and polyamine biosynthesis	0 (0.0)	3 (3.2)
Amino acid biosynthesis	14 (13.3)	8 (8.6)
Aromatic compound biosynthesis	2 (1.9)	0 (0.0)
Aminoacyl-tRNA charging	0 (0.0)	1 (1.1)
Carbohydrate biosynthesis	6 (5.7)	5 (5.4)
Cell structure biosynthesis	4 (3.8)	1 (1.1)
Cofactor, carrier, and vitamin biosynthesis	17 (16.2)	13 (14.0)
Fatty acid and lipid biosynthesis	1 (1.0)	3 (3.2)
Metabolic regulator biosynthesis	1 (1.0)	0 (0.0)
Nucleoside and nucleotide biosynthesis	19 (18.1)	3 (3.2)
Other biosynthesis	0 (0.0)	3 (3.2)
Polyprenyl biosynthesis	0 (0.0)	1 (1.1)
Secondary metabolite biosynthesis	0 (0.0)	2 (2.2)
Tetrapyrrole biosynthesis	0 (0.0)	3 (3.2)
**Superclasses:** Degradation/Utilization/Assimilation		
Alcohol degradation	0 (0.0)	1 (1.1)
Amine and polyamine degradation	2 (1.9)	2 (2.2)
Amino acid degradation	4 (3.8)	2 (2.2)
Aromatic compound degradation	8 (7.6)	5 (5.4)
Carbohydrate degradation	2 (1.9)	6 (6.5)
Carboxyrate degradation	1 (1.0)	3 (3.2)
Fatty acid and lipid degradation	1 (1.0)	0 (0.0)
Inorganic nutrient metabolism	4 (3.8)	3 (3.2)
Nucleoside and nucleotide degradation	4 (3.8)	1 (1.1)
Secondary metabolite degradation	5 (4.8)	3 (3.2)
**Superclasses:** Generation of precursor metabolites and energy		
Fermentation	2 (1.9)	7 (7.5)
Glycolysis	0 (0.0)	3 (3.2)
Pentose phosphate pathways	1 (1.0)	0 (0.0)
Photosynthesis	1 (1.0)	0 (0.0)
TCA cycle	3 (2.9)	3 (3.2)
Unassigned	1 (1.0)	3 (3.2)
**Superclasses:** Macromolecule modification		
Nucleic acid processing	1 (1.0)	1 (1.1)
**Superclasses:** Superpathways		
Unassigned	1 (1.0)	3 (3.2)

[a] Numbers in parentheses indicate the percentage of the bacterial metabolic pathways in each class among the total pathways significantly more abundant on adaxial versus abaxial leaf surfaces.

References

Bellemain E, Carlsen T, Brochmann C, Coissac E, Taberlet P, Kauserud H. 2010. ITS as an environmental DNA barcode for fungi: an in silico approach reveals potential PCR biases. BMC Microbiology **10**, 189.

Deng M, Jiang X-L, Song Y-G, Coombes A, Yang X-R, Xiong Y-S, Li Q-S. 2017. Leaf epidermal features of *Quercus* Group Ilex (Fagaceae) and their application to species identification. Review of Palaeobotany and Palynology **237**, 10-36.

Hardin JW. 1976. Terminology and classification of *Quercus* trichomes. Journal of the Elisha Mitchell Scientific Society **92**, 151-161.

Jia J, Jin P, Wu J, Wang Z, Sun B. 2015. *Quercus* (subg. *Cyclobalanopsis*) leaf and cupule species in the late Miocene of eastern China and their paleoclimatic significance. Review of Palaeobotany and Palynology **219**, 132-146.

Kim S-W, Suda W, Kim S, Oshima K, Fukuda S, Ohno H, Morita H, Hattori M. 2013. Robustness of gut microbiota of healthy adults in response to probiotic intervention revealed by high-throughput pyrosequencing. DNA Research **20**, 241–253

Liu MQ, Deng M, Zhou ZK. 2009. Taxonomic and ecological implications of leaf cuticular morphology in *Castanopsis*, *Castanea*, and *Chrysolepis*. Plant Systematics and Evolution **283**, 111-123.

Sandhu GS, Kline BC, Stockman L, Roberts GD. 1995. Molecular probes for diagnosis of fungal infections. Journal of Clinical Microbiology **33**, 2913–2919.

Simpson MG. 2010. Plant morphology. Plant systematics (Second Edition), Academic Press, 451-513.

Toyoda A, Shibata Y, Matsuo Y, *et al*. 2023. Diversity and compositional differences of the airborne microbiome in a biophilic indoor environment. Scientific Reports **13**, 8179.

Tschan GF, Denk T. 2012. Trichome types, foliar indumentum and epicuticular wax in the Mediterranean gall oaks, *Quercus* subsection *Galliferae* (Fagaceae): implications for taxonomy, ecology and evolution. Botanical Journal of the Linnean Society **169**, 611-644.
